# The Co-Inoculation Effect on *Triticum aestivum* Growth with Synthetic Microbial Communities (SynComs) and Their Potential in Agrobiotechnology

**DOI:** 10.3390/plants13121716

**Published:** 2024-06-20

**Authors:** Raimonda Mažylytė, Jurgita Kailiuvienė, Edita Mažonienė, Liana Orola, Justina Kaziūnienė, Kamilė Mažylytė, Eglė Lastauskienė, Audrius Gegeckas

**Affiliations:** 1 Life Sciences Center, Institute of Biosciences, Vilnius University, LT-10257 Vilnius, Lithuania; kamile.mazylyte@gmc.stud.vu.lt (K.M.); egle.lastauskiene@gf.vu.lt (E.L.); audrius.gegeckas@gf.vu.lt (A.G.); 2 Roquette Amilina, LT-35101 Panevezys, Lithuania; jurgita.kailiuviene@roquette.com (J.K.); edita.mazoniene@roquette.com (E.M.); 3 Faculty of Chemistry, University of Latvia, LV-1004 Riga, Latvia; liana.orola@lu.lv; 4 Institute of Agriculture, Lithuanian Research Centre for Agriculture and Forestry, LT-58344 Akademija, Lithuania; justina.kaziuniene@lammc.lt

**Keywords:** rock phosphate, organic acids, plant hormones, agrobiotechnology, synthetic community, *Triticum aestivum*

## Abstract

The use of rhizospheric SynComs can be a new and sustainable strategy in the agrobiotechnology sector. The objective of this study was to create the most appropriate SynCom composition; examine the ability to dissolve natural rock phosphate (RP) from Morocco in liquid-modified NBRIP medium; determine organic acids, and phytohormones; and verify plant growth promoting and nutrition uptake effect in the pot experiments of winter wheat (*Triticum aestivum*). A total of nine different microorganisms were isolated, which belonged to three different genera: *Bacillus*, *Pseudomonas*, and *Streptomyces*. Out of the 21 treatments tested, four SynComs had the best phosphate-dissolving properties: IJAK-27+44+91 (129.17 mg L^−1^), IIBEI-32+40 (90.95 µg mL^−1^), IIIDEG-45+41 (122.78 mg L^−1^), and IIIDEG-45+41+72 (120.78 mg L^−1^). We demonstrate that these SynComs are capable of producing lactic, acetic, gluconic, malic, oxalic, citric acids, and phytohormones such as indole-3-acetic acid, zeatin, gibberellic acid, and abscisic acid. In pot experiments with winter wheat, we also demonstrated that the designed SynComs were able to effectively colonize the plant root rhizosphere and contributed to more abundant plant growth characteristics and nutrient uptake as uninoculated treatment or uninoculated treatment with superphosphate (NPK 0-19-0). The obtained results show that the SynCom compositions of IJAK-27+44+91, IIBEI-32+40, IIIDEG-45+41, and IIIDEG-45+41+72 can be considered as promising candidates for developing biofertilizers to facilitate P absorption and increase plant nutrition.

## 1. Introduction

Plants create interactions with microorganisms around and inside their roots [1]. These interactions of root-associated microorganisms are considered to be the root microbiota [2]. The root microbiota is essential to plant health, growth, and development and plays an important role by modulating plant hormone homeostasis, promoting nutrient acquisition, and improving resilience to abiotic or biotic stresses [3,4].

As technology advances, it becomes increasingly important to apply organic farming methods [5]. One important method is the application of beneficial microorganisms into agricultural soils. For the past few decades, microorganisms with plant growth-promoting (PGP) traits were isolated and used as inoculants to improve crop health and production. The PGP microorganisms assist plant growth by enhancing nutrient acquisition such as phosphorus and potassium solubilization, nitrogen fixation, ammonia (NH_3_), hydrogen cyanide (HCN), 1-aminocyclopropane-1-carboxylate (ACC) deaminase, and siderophore production, and producing plant growth-promoting substances such as phytohormones, enzymes, antibiotics, or ability to induce plant resistance [6]. Most research focuses on single microorganism application to plants grown as biofertilizers and it should be noted that it happens essentially in sterile conditions [7,8]. This means that the microorganism must enter the local microbiota which naturally implies a non-sterile environment. Competition with local microbiota has a huge impact and the adaptation of a single microorganism into the plant rhizosphere can be unsuccessful [9]. For inoculums to be successful in field trials, in-depth knowledge of microbial diversity and abundance in the plant rhizosphere and microorganisms’ interactions with plant host is necessary [10].

Nevertheless, an alternative could be to create synthetic microbial communities (SynComs), because this community has a better chance to survive and function in a non-sterile environment [11,12]. A favorable way to defeat the challenges associated with natural communities is to design synthetic communities by co-culturing multiple taxa under strictly defined conditions to simulate the structure and function of a real plant microbiota. The formation of effective SynComs consists of an opportunity to identify the core microorganisms associated with plants. Though, the rate and ability of SynCom to colonize and interact with indigenous soil microbiota are mostly unknown [13]. An appropriate inoculant must be able to compete with other microorganisms, efficiently colonize plant roots, and demonstrate a strong association with plants throughout the growing season [11]. Even though the application of SynCom for crop production is in its infant stage, advances in technologies are occurring at a remarkable pace and it is an approach that has the potential to deliver a solution in the quest toward sustainable agriculture. SynComs consist of carefully chosen microorganism species to produce the desired microbiota composition [12].

Many microorganisms can influence plant growth characteristics through some direct and indirect pathways [14]. Direct microbial mechanisms involved the production of plant growth-promoting (PGP) substances such as organic acids and phytohormones as well as the solubilization of mineral phosphates, reducing the need for phosphorus mineral fertilizer in soils. Indirect microbial mechanisms include preventing the deleterious effects of the pathogens in soils by producing antifungal or antibacterial compounds (e.g., siderophores and enzymes for pathogen cell wall-degrading effect) [15,16,17]. Research of the last decade also revealed that the composition, diversity, metabolites of SynCom, and their interactions with indigenous plant microbiota prominently influence the plant immune system [18].

Also, in recent decades, phosphorus in an available form to plants was a particularly relevant topic [19]. The soils contain a lot of various phosphorus compounds which plants cannot use for their own vegetative processes [20]. Phosphorus, in the form of natural rocks, occurs naturally in soil and is a critical nutrient for plant growth [21]. Phosphorus is an essential macronutrient required for biochemical processes such as photosynthesis, energy storage, signal and energy transfer, cell division, nitrogen fixation, and respiration [22,23]. The availability of nutrients in soils for plant uptake is limited by several soil factors [24]. It is very important to emphasize that one such factor is soil pH levels [25]. However, the availability of phosphorus for plant uptake can be managed by the application of organic amendments for acidic and alkaline soils and by the regulation method of P biofertilizer application, and from the point of view of organic farming, the use of microbiological fertilizers is especially attractive in this regard [26,27,28].

Consequently, the aim of this research was to isolate *Triticum aestivum* (*ARTIST C2* variety; Deutsche Saatveredelung, Germany) rhizosphere microorganisms from three different agricultural fields with the same crop rotation technology which belong to neutral (pH 7.08), slightly acidic (pH 6.11) and slightly alkaline (pH 8.00) soils and select three isolates of different genera for each treatment to compose the most suitable SynCom for P-solubilizing. Also, we ascertain the ability to produce lactic, acetic, gluconic, malic, oxalic, and citric acids and phytohormones such as indole-3-acetic acid, zeatin, gibberellic acid, and abscisic acid. The aim was to determine the potency of the best composition SynComs to increase *Triticum aestivum* growth parameters and the nutritional composition of the absorption.

## 2. Results and Discussion

### 2.1. Experimental Area and Soil Analysis

An experiment of all the agricultural fields was performed at the research farm of the ZUB Krekenava, Pusalotas region located in Panevezys, and the geographical coordinates and altitude of these areas are defined below. The climate type of the experimental areas is classified as *Dfb* according to Köppen–Geiger classification [29]. The evaluated main climatic characteristics are as follows: the average annual lower temperature was −6.0 °C; the average annual higher temperature was 23.0 °C; the average annual rainfall was 65.1 mm and all the data are summarized in Table 1.

Composite soil samples were collected from the experimental sites in the soil layer of 0–0.20 cm depth for the chemical analysis. The information about each agricultural field’s chemical analyses are presented in Table 2.

### 2.2. Identification and Characterization of PGPR Isolates

**Figure 1:**
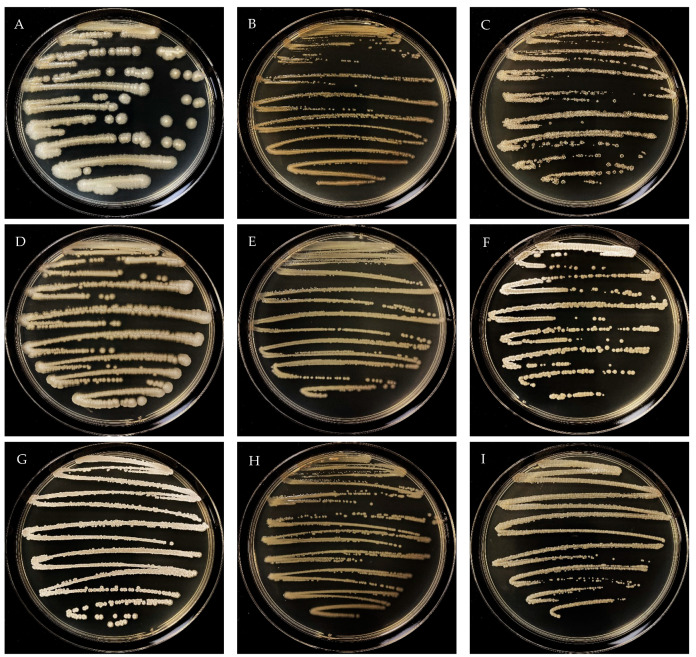
Colony morphology of *Bacillus toyonensis* IJAK-27 (**A**); *Pseudomonas bijieensis* IJAK-44 (**B**); *Streptomyces gardneri* IJAK-91 (**C**); *Bacillus aryabhattai* IIBEI-32 (**D**); *Pseudomonas helmanticensis* IIBEI-40 (**E**); *Streptomyces anulatus* IIBEI-22 (**F**); *Bacillus tequilensis* IIIDEG-45 (**G**); *Pseudomonas granadensis* IIIDEG-41 (**H**); and *Streptomyces badius* IIIDEG-72 (**I**).

In this study, a total of nine different microorganisms were isolated, three isolates from each agricultural field (three fields × three isolates = nine different microorganisms) (Figure 1). The 16S rDNA sequence analysis assigned them to the three genera *Bacillus*, *Pseudomonas*, and *Streptomyces*, respectively. The isolates IJAK-27, IJAK-44, and IJAK-91 shared 16S rDNA similarity with *Bacillus toyonensis* BCT-7112 (100%), *Pseudomonas bijieensis* L22-9 (100%), and *Streptomyces gardneri* NBRC 12865 (100%), respectively. The isolates IIBEI-32, IIBEI-40, and IIBEI-22 were closely related to *Bacillus aryabhattai* B8W22 (100%), *Pseudomonas helmanticensis* OHA11 (100%), and *Streptomyces anulatus* NRRL B-2000 (100%), respectively. The isolates IIIDEG-45, IIIDEG-41, and IIIDEG-72 were most closely related to *Bacillus tequilensis* KCTC 13622 (100%), *Pseudomonas granadensis* LMG 27940 (100%), and *Streptomyces badius* NRRL B-2567 (100%), respectively. The molecular identification information of PGPR by 16S rDNA sequencing is submitted in Table 3.

**Figure 2:**
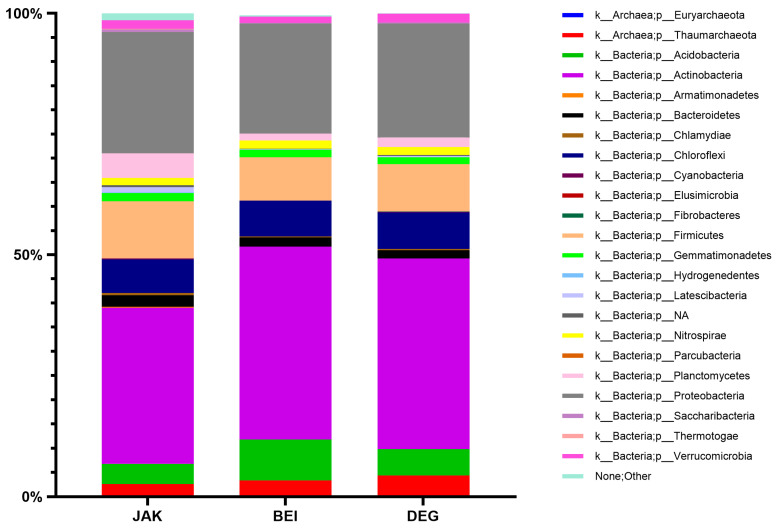
Relative abundance of the different dominated phyla in the studied soils.

Among the isolated PGPR, at the phylum level, *Actinobacteria* was the dominant phylum in JAK (32.2%), BEI (39.9%), and DEG (39.4%) soils. Next, the second phylum *Proteobacteria* was dominated in JAK (25.1%), BEI (22.8%), and DEG (23.6%) soils. While *Firmicutes* was the third-level phylum, it also dominated in JAK (11.9%), BEI (9.0%), and DEG (9.8%) rhizosphere soil samples. The composition of the other phyla in JAK, BEI, and DEG rhizosphere soil samples accounted for 30.8%, 28.3%, and 27.2%, respectively, of the remaining amount (Figure 2).

**Figure 3:**
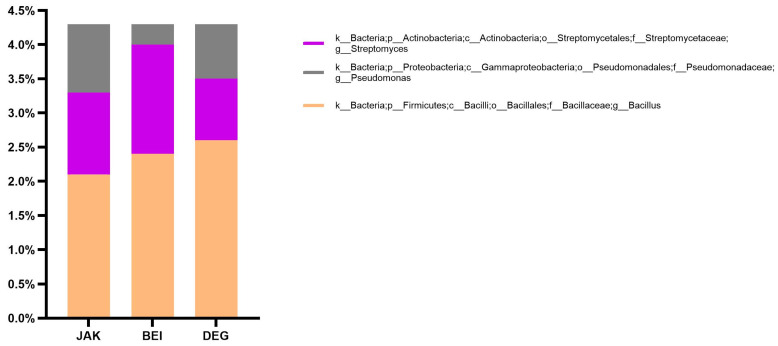
Relative abundance of the different dominated genera of PGPR in the studied soils.

At the genus level, *Bacillus* was the most dominant genus of the *Bacillaceae* family in the JAK (2.1%), BEI (2.4%), and DEG (2.6%) rhizosphere soil samples. Although the *Bacillus* belonged to the *Firmicutes* phyla, at the genus level it accounted for the highest amounts. However, *Actinobacteria* was the most dominant phyla, but at the genus level, *Streptomyces* was relatively less compared to the genus of *Bacillus*. The *Streptomyces* was the supreme dominant genus of the *Streptomycetaceae* family, *Actinobacteria* phylum in JAK (1.2%), BEI (1.6%), and DEG (0.9%). In these analyses, the *Pseudomonas* genus of the *Pseudomonadaceae* family showed lower abundances. Nevertheless, the *Proteobacteria* phylum was the second most abundant phylum in the samples. Accordingly, the genus of the *Pseudomonas* was determined in JAK (1.0%), BEI (0.3%), and DEG (0.8%) (Figure 3).

The rhizosphere soil microbiota contains a wide variety of various microorganisms, which play vital roles in soil nutrition and plant growth promotion [30]. Actually, the dominant bacterial phyla in most soil samples include *Proteobacteria*, *Actinobacteria*, *Acidobacteria*, *Firmicutes*, *Planctomycetes*, *Chlorflexi*, and *Verrucomicrobia* [31]. At the genera level, similar results were obtained by Janssen P.H., stating that the genera *Agrobacterium*, *Alcaligenes*, *Arthrobacter*, *Bacillus*, *Flavobacterium*, *Micromonospora*, *Nocardia*, *Pseudomonas*, and *Streptomyces* were estimated as the dominant genera in soils [32]. The dominant composition of bacterial phyla of microbiota in soil is significantly different on many factors [33]. Various environmental factors, such as temperature, soil pH and humidity, geographical location, climate type, cultivated plant culture, and crop rotation were proved to affect the architecture of the soil microbiota [34,35]. In recent decades, the structure, diversity, and activity of soil microbiota communities were progressively used as the indicators of soil health and yield potential [36,37]. Determining changes in soil microbiota communities is necessary to plant health and productivity, and improves the variety and stability of agroecosystems [38,39].

The entire PGPR and the characteristics demonstrated by all the isolates are described in Table 4. The isolates were found to be fast growers (1–2 days of incubation period). Out of the nine morphotypes obtained, seven of the colonies showed positive results of NH3 production by colorimetric detection. The isolates IJAK-27, IIBEI-32, and IIIDEG-45 were the maximum producer of NH3 among the nine isolates. The highest HCN production was detected by the isolates IJAK-27 and IIIDEG-45, and the color change was not found in the HCN detection experiment performed for IIBEI-22 and IIIDEG-72, which indicated that IIBEI-22 and IIIDEG-72 are negative HCN producers. For a siderophore production experiment, a yellow-colored halo around the bacterial colonies grown in CAS agar plates was observed for all nine isolates. This result indicates that all the isolates can produce siderophores, especially IJAK-44 and IIIDEG-41 isolates. However, all the bacterial isolates showed positive results for ACC deaminase production. The isolated bacteria are also tested by catalase activity. While a vivid appearance of oxygen bubbles was observed for IJAK-27 and IIIDEG-45, the bubble was comparatively less for IJAK-91, IIBEI-40, IIBEI-22, and IIIDEG-72. A total of nine distinct colonies were selected and designated as nitrogen-fixing bacteria. The isolate IIIDEG-45 showed luxuriant growth on nitrogen-free agar plate (0.5% BTB) and it intensified the blue color of BTB around the colony. The IJAK-44 and IIIDEG-41 strains exhibited clear zones of potassium solubilization with yellow color formation due to acid production surrounding the colony when grown on Aleksandrow Agar medium with added BTB as a pH indicator. After 7 days of incubation period, all the PGPR isolates were screened for phosphate solubilization activity using Pikovskaya’s agar medium where tricalcium phosphate was used as the sole carbon source. A clear halo of phosphate solubilization around the colonies was observed and indicated as a positive result.

### 2.3. Phosphorus in Liquid NBRIP Medium with RP and Organic Acid Detection

**Figure 4:**
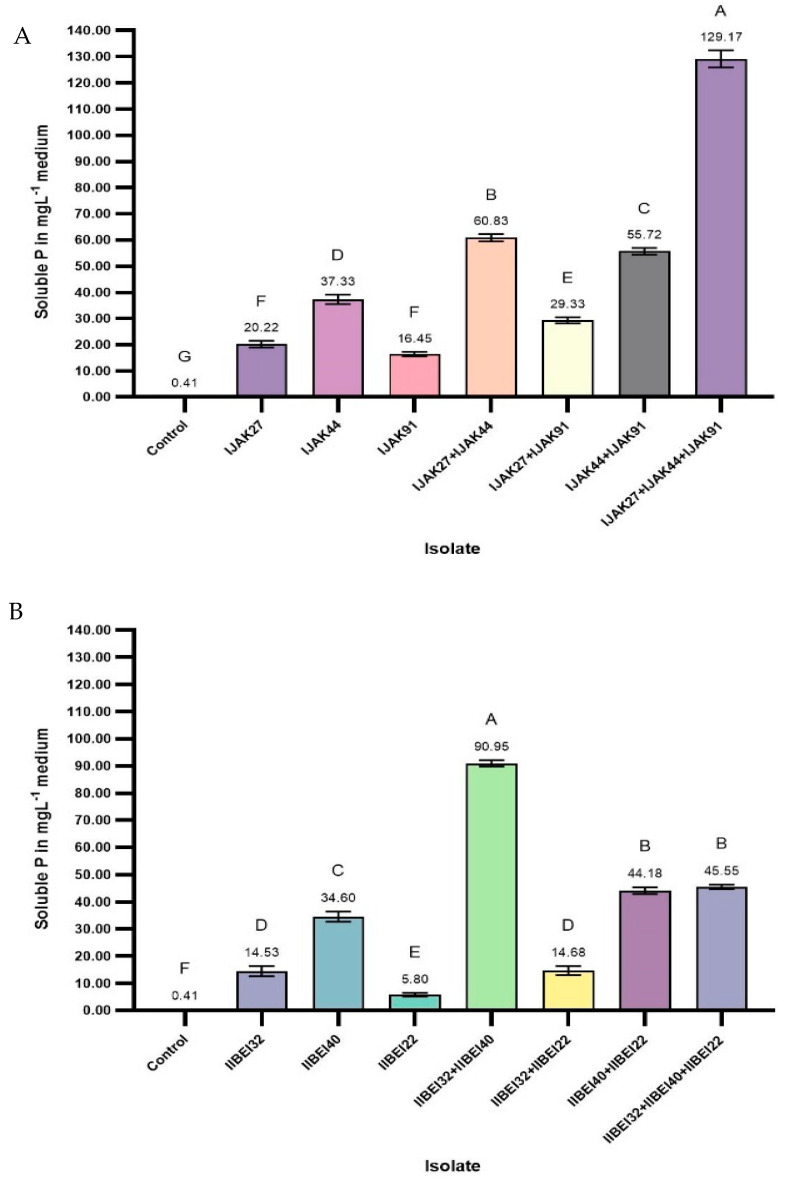

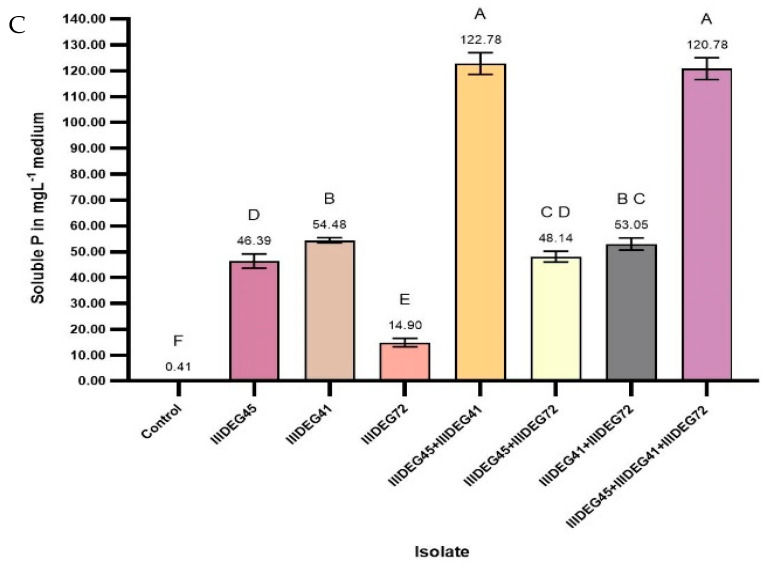
The influence of PGPR co-inoculation on phosphate uptake was tested in NBRIP liquid media to investigate the PGPR interaction efficiency in remediating the combined phosphorus solubility in soil. Although the solubilization levels in the liquid media varied among different isolates, all the isolates could solubilize rock phosphate as the only source of phosphate in the NBRIP liquid media (**A**–**C**). The differences between the mean solubilization ability in the culture medium are statistically significant according to the one-way ANOVA (*p* < 0.05). The vertical bars represent confidence intervals at a confidence level of 95% (n = 3). The same letters indicate that differences among the means are not statistically significant (Tukey’s HSD post hoc test).

On the first attempt, the three isolates isolated from the JAK agricultural field were used for seven different treatments using these microorganisms for sole inoculation or co-inoculation in equal parts. The phosphate solubilization ability of the selected isolates’ sole inoculation or co-inoculation in NBRIP liquid medium, with natural RP from Morocco as the only source of insoluble phosphorus, varied from 16.45 to 129.17 mg L^−1^. The co-inoculation with IJAK-27+IJAK-44+IJAK-91 produced the highest concentration of soluble phosphate in the NBRIP broth after 30 days of incubation (129.17 mg L^−1^). The co-inoculation of IJAK-27+IJAK44 and IJAK-44+IJAK-91 also exhibited a high level of phosphate solubilization capability in the liquid NBRIP. The values obtained were 60.83 mg L^−1^ for IJAK-27+IJAK-44 and 55.72 mg L^−1^ for IJAK-44+IJAK-91. However, the sole inoculation of IJAK-44 showed better phosphate solubilization results (37.33 mg L^−1^) than the co-inoculation of IJAK-27+IJAK-91 (29.33 mg L^−1^). Finally, sole inoculation IJAK-27 and sole inoculation IJAK-91 showed statistically lower solubility compared with the other treatments (20.22 mg L^−1^ and 16.45 mg L^−1^, respectively) (Figure 4A).

In the second research, the three isolates isolated from the BEI agricultural field showed different solubility abilities compared to the first attempt. The solubilization of RP in the NBRIP liquid medium varied from 5.80 to 90.95 µg mL^−1^, and the co-inoculation of IIBEI-32+IIBEI-40 demonstrated the maximal capability. Next, two other co-inoculations, IIBEI-32+IIBEI-40+IIBEI-22 and IIBEI-40+IIBEI-22, demonstrated the highest results in this co-inoculation group of microorganisms. However, statistically significant differences were not found in both the treatments (45.55 mg L^−1^ and 44.18 mg L^−1^, respectively). The single inoculation of IIBEI-40 had a significantly higher phosphate solubilization capability (34.60 mg L^−1^) than the other three inoculations. The treatments IIBEI-32+IIBEI22 and IIBEI-32 also showed a slightly significant difference in phosphate solubilization concentration (14.68 mg L^−1^ and 14.53 mg L^−1^, respectively). On the contrary, the treatment IIBEI-22 had the lowest possibility of the dissolution of an insoluble phosphorus compound (5.80 mg L^−1^), suggesting that IIBEI-22 cannot be used as a phosphate-solubilizing microorganism (Figure 4B).

In the last attempt, the RP solubility concentrations ranged from 14.90 to 122.78 mg L^−1^. In this treatment group, two co-inoculation options, IIIDEG-45+IIIDEG-41 and IIIDEG-45+IIIDEG-41+IIIDEG-72, observed the best results with no statistical difference (122.78 mg L^−1^ and 120.78 mg L^−1^, respectively). The sole inoculation of IIIDEG-41 and co-inoculation of IIIDEG-41+IIIDEG-72 showed a marginally significant difference (54.48 mg L^−1^ and 53.05 mg L^−1^, respectively). However, co-inoculation with IIIDEG-41+IIIDEG-72 also showed a slight change compared to IIIDEG-45+IIIDEG-72 (48.14 mg L^−1^). On the other hand, no difference was observed between co-inoculation with IIIDEG-45+IIIDEG-72 and single inoculation with IIIDEG-45 (46.39 mg L^−1^). Moreover, IIIDEG-72 showed the statistically lowest solubility (14.90 mg L^−1^) (Figure 4C).

The organic acids were determined in the culture supernatant of single inoculation or co-inoculation in the NBRIP medium without RP at 30 °C after 48 h. Based on Nautiyal et al.’s [40] results, liquid NBRIP medium was selected for analysis, which was classified as the best medium for testing the phosphate solubilization. Six different organic acids, including lactic, acetic, gluconic, malic, oxalic, and citric acids, were detected. A sterile NBRIP medium without RP was used as the control sample. Of the six different organic acids, lactic acid was detected in the largest quantity (567.0 µg mL^−1^) in the co-inoculation of IJAK-27+IJAK-44+IJAK-91, followed by IIIDEG-45+41 and IIIDEG-45+41+72 (455.0 ± 15.0 and 405.0 ± 7.0, respectively). The maximum concentration of acetic acid was established in the co-inoculation of IJAK-27+44+91, IIIDEG-45+41, and IIIDEG-41+72 without significant statistical difference (35.0 ± 2.0, 35.0 ± 2.0, and 34.0 ± 2.0, respectively). The co-inoculation of IIBEI-32+40, IIBEI-40+22, and IIBEI-32+40+22 was characterized by the best gluconic acid synthesis compared to other treatment options (180.0 ± 7.0, 140.0 ± 4.0, and 139.0 ± 7.0, respectively). Three treatments, IJAK-27+44+91, IIBEI-32+40, and IIBEI-40+22, produced the highest concentration of malic acid (140.0 ± 7.0, 105.0 ± 5.0, and 92.0 ± 2.0, respectively). Oxalic acid had the lowest concentration in all the treatment variants. The co-inoculation of IJAK-27+44, IIBEI-40+22, and IIIDEG-45+41+72 showed the best results, although the concentrations were lower than other detected acids (1.8 ± 0.2, 1.5 ± 0.2, and 1.3 ± 0.1, respectively). The highest citric acid concentrations were determined in the co-inoculation of IJAK-27+44, IJAK-27+44+91, and IIBEI-32+40 (70.0 ± 4.0, 33.5 ± 6.0, and 27.0 ± 0.2, respectively) (Table 5).

In general, this research showed that the different rhizobacteria isolates or isolated consortiums tested have different abilities to solubilize P in a liquid medium. It may be due to the different abilities of the isolates to produce and secrete organic acids among these isolates. The highest concentrations of these organic acids were detected in the double or triple co-inoculations of the isolated microorganism SynComs. In summary, our results revealed that genus *Bacillus*, *Pseudomonas*, and *Streptomyces* can dissolve insoluble phosphorus compounds in soils. However, to obtain an optimal result, it is necessary to conduct more detailed studies and achieve the best treatment variant. In the continuation, the consortiums of selected microorganisms were analyzed to determine phytohormones and their concentrations by Liquid Chromatography Time-of-Flight Mass Spectrometry. Four communities of microorganisms which were superior in the solubility of the insoluble phosphorus compound from the experiment described above were selected for further and more detailed analysis. Also, it was decided to conduct pot experiments simulating field conditions to find out the association between organic acid synthesis, phytohormones, and plant productivity.

### 2.4. Analysis of Phytohormones by Liquid Chromatography Time-of-Flight Mass Spectrometry (LC-TOF/MS)

The Liquid Chromatography Time-of-Flight Mass Spectrometry was used to analyze phytohormones in the selected samples for further investigation. Four different plant hormones, including indole-3-acetic, zeatin, gibberellic acid, and abscisic acid were detected in the bacterial supernatants of the four consortiums composed of screened microorganisms. A sterile NBRIP medium was used as the control sample. Of the different plant hormones, indole-3-acetic acid was identified in the largest quantity (6.600 ± 0.500 µg mL^−1^) in the treatment IJAK-27+44+91, followed by the IIBEI-32+40 (3.500 ± 0.300 µg mL^−1^), IIIDEG-45+41 (0.382 ± 0.005 µg mL^−1^), and IIIDEG-45+41+72 (0.348 ± 0.020 µg mL^−1^) treatments. The highest concentration of zeatin was determined in the co-inoculation of IIIDEG-45+41 (1.100 ± 0.200 µg mL^−1^), followed by IIBEI-32+40 (0.160 ± 0.010 µg mL^−1^), IIIDEG-45+41+72 (0.159 ± 0.011 µg mL^−1^), and IJAK-27+44+91 (0.150 ± 0.020 µg mL^−1^). The co-inoculation of IJAK-27+44+91 showed the best concentration (3.400 ± 0.400 µg mL^−1^), and lower concentrations were detected in IIIDEG-45+41+72 (2.250 ± 0.040 µg mL^−1^), IIIDEG-45+41 (1.700 ± 0.300 µg mL^−1^), and IIBEI-32+40 (1.240 ± 0.100 µg mL^−1^). The maximum amount of abscisic acid was detected in the co-inoculation of IIIDEG-45+41 and IIIDEG-45+41+72 (0.271 ± 0.006 and 0.271 ± 0.012, respectively), and lower concentrations detected in the co-inoculation of IJAK-27+44+91 and IIBEI-32+40 (0.250 ± 0.020 and 0.136 ± 0.003, respectively) (Table 6).

### 2.5. Co-Inoculation Test in Sterile Soils through Pot Experiment

The co-inoculation of the PGPR strains in most cases increased the growth indexes of *T. aestivum* compared to the control samples. Two control samples were selected: CON1 (the sample without co-inoculation and mineral P fertilizers) and CON2 (the sample without co-inoculation and with granular superphosphate (NPK 0-19-0) applied as 200–300 Kg ha^−1^ according to the manufacturer’s specified rate).

Co-inoculation with the PGPR strains increased the root and shoot lengths compared to the CON1 and CON2 treatments. The co-inoculation of IJAK-27+44+91 recorded the highest root (36.00 cm) and shoot lengths (63.50 cm) compared to IIBEI-32+40 root (35.17 cm) and shoot lengths (63.17 cm), followed by IIIDEG-45+41 root (34.33 cm) and shoot lengths (62.00 cm) and IIIDEG-45+41+72 root (33.83 cm) and shoot lengths (61.50 cm). However, inoculation with IIIDEG-45+41 and IIIDEG-45+41+72 recorded significantly higher root and shoot lengths compared to the mineral P fertilizer application (27.33 and 55.83 cm, respectively) and control (25.83 and 51.67 cm, respectively). It can be concluded that different PGPRs vary in their ability to increase growth indexes when co-inoculated together and show a higher response than fertilizing only with mineral fertilizers or without using any fertilization technology.

In terms of the number of leaves, there was no significant difference between the co-inoculation of IJAK-27+44+91 and the co-inoculation of IIBEI-32+40 (8.30 and 7.96 number of leaves, respectively). Also, IIIDEG-45+41, IIIDEG-45+41+72, and CON2 showed no significant difference between each other (7.86, 7.90, and 7.90 number of leaves, respectively). However, the CON1 of winter wheat recorded significantly less number of leaves compared to all the treatments (6.28 number of leaves).

Next, the co-inoculation of IIBEI-32+40 and IJAK-27+44+91 resulted in higher shoot fresh weight compared to the other treatments (9.62 and 9.60 g, respectively). Similarly, the co-inoculated plants with IJAK-27+44+91 and IIBEI-32+40 resulted in higher root fresh weight (10.93 and 10.73 g, respectively) compared to the other two co-inoculations of IIIDEG-45+41 or IIIDEG-45+41+72 (8.23 and 7.85 g, respectively, of the shoot fresh weight; 8.85 and 8.80 g, respectively, of the root fresh weight) and CON2 and CON1 (6.92 and 6.15 g, respectively, of the shoot fresh weight; 8.68 and 7.05 g, respectively, of the root fresh weight). However, the analysis did not show any differences between the inoculation of winter wheat with IJAK-27+44+91 and IIBEI-32+40 in root fresh weight. Similarly, no differences were observed between the co-inoculation of IIIDEG-45+41 and IIIDEG-45+41+72. All the parameters measured showed that the two co-inoculation treatments had an optimum effect on the shoot and root fresh weights of winter wheat.

Further on, the co-inoculation of IJAK-27+44+91 recorded a higher root dry weight as compared to the IIBEI-32+40 co-inoculation. For instance, IJAK-27+44+91 recorded a significantly (α = 0.05) higher root dry weight (0.97 g) compared to IIBEI-32+40 (0.89 g). Similarly, there was a significant difference between the IIIDEG-45+41 and IIIDEG-45+41+72 co-inoculations of root dry weights (0.83 and 0.77, respectively). However, the combination of IIBEI-32+40 led to significantly higher shoot dry weight (0.90 g) compared to IJAK-27+44+91 (0.87 g), but no significant difference was observed between co-inoculated with IIIDEG-45+41 (0.71 g) or IIIDEG-45+41+72 (0.69 g). However, all four treatments led to a significant increase in root and shoot dry weights over the CON1 or CON2. Mineral phosphorus application CON2 recorded the highest root and shoot dry weights (0.66 and 0.57 g, respectively) than CON1 (0.54 and 0.53 g, respectively) (Table 7).

Further analyses were carried out to evaluate plant growth indexes: sugars, pH, EC, K, Ca, Mg, Na, NH_4,_ NO_3,_ N (in nitrate), N (total nitrogen), Cl, S, P, Si, Fe, Mn, Zn, B, Cu, Mo, and Al contents. The highest Mg (427 ppm), S (208 ppm), P (638 ppm), Mn (7.64 ppm), and B (2.60 ppm) contents were recorded in the IJAK-27+44+91 treatment. The IIBEI-32+40 treatment increased the Si (26.3 ppm), and Mo (0.10 ppm) contents. Moreover, higher K (4879 ppm), NH_4_ (57 ppm), N (total nitrogen) (1975 ppm), Fe (2.75 ppm), Zn (3.6 ppm), Mo (0.10 ppm), and Al (1.05 ppm) contents were recorded in IIIDEG-45+41. IIIDEG-45+41+72 excelled at the highest NO_3_ (166 ppm), N (in nitrate) (38 ppm), and Cu (0.37 ppm) contents_._

Compared with the CON2, the co-inoculation of the IJAK-27+44+91 treatment increased the K (4470 ppm), Ca (1390 ppm), Mg (427 ppm), NH_4_ (52 ppm), S (208 ppm), P (638 ppm), Si (23.8 ppm), Fe (2.21 ppm), Mn (7.64 ppm), and B (2.60 ppm) contents of winter wheat plant. Co-inoculation with IIBEI-32+40 showed a profound increment in most of the nutrients, with significantly higher Ca (1634 ppm), Mg (410 ppm), S (191 ppm), P (635 ppm), Si (26.3 ppm), Fe (2.7 ppm), Zn (2.3 ppm), B (2.4 ppm), and Al (1.01 ppm) contents compared with the CON2 treatment. The co-inoculation of the IIIDEG-45+41 treatment also contributed to the accumulation of K (4879 ppm), Ca (1112 ppm), NH_4_ (57 ppm), N (total nitrogen) (1975 ppm), S (182 ppm), Si (19.9 ppm), Fe (2.75 ppm), Zn (3.6 ppm), B (2.47 ppm), Cu (0.31 ppm), and Al (1.05 ppm) to a significant level compared with the CON2. The treatment IIIDEG-45+41+72 showed higher Ca (1328 ppm), NH_4_ (51 ppm), NO_3_ (166 ppm), N (in nitrate) (38 ppm), Si (19.4 ppm), Fe (2.22 ppm), Mn (7.54 ppm), Zn (2.04 ppm), B (2.36 ppm), and Cu (0.37 ppm). Among all these treatments, the CON1 treatment led to the lowest nutrient contents of winter wheat plants, no matter which element was used for comparison (Table 8).

The composition of the root-associated microbiota is designed by complex multilateral interactions between abiotic environment factors and biotic inhabitants [41]. Depending on the outcome of an interaction with the host, microorganisms are considered as mutualistic, commensal, or pathogenic [42]. Several studies have shown that bacterial communities are dynamically shaped by environmental factors such as season, soil type, soil history, nutrient content, and water content as well as host factors, especially species and developmental stage [43,44]. Microbial species richness is highest in bulk soil, decreases in the rhizosphere, and is lowest in the endophytic and phyllospheric compartments [45,46]. In parallel, microbial cell count increases from bulk soil toward the root surface, indicating favorable conditions for the selected microbial species to colonize plant roots [47]. Plants release about 10–40% of the many nutrients, especially carbon fixed by a plant, that can be released via roots into the rhizosphere; it is obvious that the plant takes an active role in shaping its own core microbiome [48]. Despite the great biodiversity of soils, the microbial community in the rhizosphere of plants is dominated by four bacterial phyla: *Actinobacteria*, *Proteobacteria*, *Firmicutes*, and *Bacteroidetes* [49].

Actually, it was found that Actinobacteria were abundant in the rhizosphere of T. aestivum [50]. Actinobacteria are the main source of bioactive compounds, such as secondary metabolites, antibiotics, and enzymes with agriculture importance known to date [51,52]. A very important genus of this phylum, such as Streptomyces, was isolated from agricultural soil sources [53]. Streptomyces spp. have potential as a component in ecological farming as biocontrol agents and biofertilizers [54]. Furthermore, some Streptomyces spp. are known as PGPRs that are able to promote P-solubilizing [55,56]. It can be noted that some species of Streptomyces released pyruvic, α-ketoglutaric, lactic, malic, citric, succinic, and oxalic organic acids [57]. Also, it directly promotes plant growth by the production of phytohormones (auxins, cytokinins and gibberellins, abscisic acid, salicylic acid, and jasmonic acid) [58,59].

Further, *Pseudomonas* spp., belonging to the phylum of *Proteobacteria*, are characterized by plant growth-stimulating properties [60]. For example, it was indicated that *Pseudomonas* putida M17 was isolated from rhizosphere soil samples and increased *Phaseolus vulgaris* nodulation compared to *Rhizobium phaseoli*. Furthermore, 2-ketogluconic acid, a phosphate-solubilizing compound, was detected in *Pseudomonas putida* M17 [61]. Also, in the next experiment determined that phytohormones-producing strain *Pseudomonas fluorescens* G20–18 enhanced tomato plant growth and boosted its tolerance to drought stress when the microorganism cell suspension was used as biocontrol fertilizers [62].

The members of the phylum *Firmicutes*, especially the genus of *Bacillus*, were the most abundant in the rhizosphere and were important for improving soil quality and consequently favoring the growth of winter wheat [63]. Generally, *Bacillus* spp. are one of the most common genera that are used in organic agriculture as a biological fertilizer [64]. The literature is full of research and analyses with *Bacillus* spp. because these species are particularly distinguished by the fact that microorganism cells are spore-forming, which facilitates cell viability and the storage of biofertilizers [65,66]. *Bacillus* spp. are widely used as PGPRs and have a very wide range of properties. It was observed that *Bacillus* species increased the production components and yield of common beans, P content in the leaves, P accumulation, number of pods per plant, dry matter, and yield. The authors indicated that P_2_O_5_ application increased yield by 79 Kg ha^−1^ for each 10 Kg ha^−1^ of P_2_O_5_ added, while inoculation with PGPR (dose of 159 mL ha^−1^) increased yield by 12% (389 Kg ha^−1^) compared to the management without inoculation [67]. *Bacillus* spp. can produce organic acids, including gluconic, acetic, lactic, malic, butyric, oxalic, succinic, and propionic acids [68,69]. Organic acid production by rhizosphere microorganisms is considered the main mechanism used by phosphate-solubilizing microorganisms to dissolve inorganic phosphate compounds [70]. Moreover, *Bacillus* spp. are characterized by phytohormones production, including auxins, cytokinins, gibberellins, and abscisic acid [71,72]. The authors indicated that *Bacillus safensis* TS3 has the ability to produce phytohormones such as indole 3-acetic acid, abscisic acid, and trans-zeatin [73]. In another scientific article, the authors noted that *Bacillus* NMCN1 and LLCG23 strains have promoted *T. aestivum* plants’ growth through the modulation of phytohormones [74].

Most biological products are composed of a single species of microorganism. The creation of complex biological fertilizers, i.e., a mixture of several genera or species of microorganisms, is a labor-intensive process [75]. Indeed, interactions between the members of the different genera range from mutualism to competition depending on media, bioactive compound potential, and phylogeny [76]. Although these interactions are tricky, their analysis and research are necessary for further ecological agricultural processes [77,78]. Thus, in one research, the authors stated that the tri-species synthetic community (SynCom) of *Bacillus cereus*, *Pseudomonas koreensis,* and *Flavobacterium johnsonii* synergistically increases biofilm formation, even though *P. koreensis* restricts the growth of *F. johnsonii*, as *B. cereus* limits the concentration of the koreenceine antibiotic from *P. koreensis* [76].

In another research, SynCom, which consisted of *Massilia* sp. SA087, *Enterobacter* sp. SA187, *Ensifer* sp. SA403, *Bacillus* sp. SA436, and *Streptomyces* sp. SA444 led to the strongest growth response to the tomato plants growing in a non-sterile substrate against high salt stress and having a 34% increase in dry shoot biomass compared to the non-inoculated control treatment [79]. SynCom structure strategy must be a combination of plant phenotype screening and more advanced and accurate methods, such as metatranscriptomic, metagenomic, and metabolomic sequencing, to better model the predictive traits for a successful SynCom design [80,81].

Further, in the following study, SynCom was constructed of eight different PGPR isolated from the *T. aestivum* rhizosphere soil samples, including three *Bacillus* spp., two *Acinebacter* spp., an *Enterobacter* sp., a *Xanthomonas* sp., and a *Burkholderia* sp., which demonstrated plant growth-promoting characteristics, including IAA and NH_3_ production, antifungal suppression, increased the plant growth, root development, and biomass production [13].

The root-associated microbiome, including plant growth-promoting rhizobacteria (PGPR), may promote plants’ improved growth and development due to the more efficient absorption of water and nutrients, and regulates plant nutrient metabolism during the entire growing season, preserves and maintains good soil structure, and regulates soil agrochemical properties, the most important of which are soil pH level, organic matter, and mobile macro- and microelement quantities. It is essential to carry out research and analysis in order to design the best composition of SynCom with its best properties [82,83].

In this study, the new SynCom constructions of various *Bacillus* spp., *Pseudomonas* spp., and *Streptomyces* spp. showed important results. Recent research suggests that the microbiome community can be altered via biocontrol inoculation to support specific taxa in the rhizosphere. *Bacillus* spp., *Pseudomonas* spp., and *Streptomyces* spp. have been reported as effective P-solubilizers, able to release organic acids, produce phytohormones, and promote plant growth and nutrient uptake. The application of IJAK-27+44+91, IIBEI-32+40, IIIDEG-45+41, and IIIDEG-45+41+72 SynCom as a biofertilizer could be an alternative option to reduce considerable amounts of mineral phosphorus fertilizers, increase soil biological activity, protect plants from diseases and pathogens, and promote plant growth, crop yields, and production quality. Although the IJAK-27+44+91 treatment performed best in P-solubilization, all four selected variants had positive properties as PGPR and demonstrated strong application potential and probable utility for future agricultural and biotechnological applications. In the future, experiments and analyses should be conducted in the soil to validate the effective capability of those PGPR in real field conditions and select the most suitable SynCom according to the plant culture and the chemical and physical properties of the soil.

## 3. Materials and Methods

### 3.1. Sample Location

The research was performed using soils from three different locations in which the same crop rotation technology was applied. The *T. aestivum* (*ARTIST C2* variety; Deutsche Saatveredelung, Germany) was cultivated in neutral (pH 7.08), slightly acidic (pH 6.11), and slightly alkaline (pH 8.00) plantations. The bulk and rhizosphere soil samples were collected in the month of July 2022 from the *T. aestivum* agricultural fields of Jakuboniai (55°54′00.3″ N 24°06′44.3″ E), Beicarke (55°55′53.6″ N 24°16′46.2″ E), and Deglenai (55°56′17.8″ N 24°08′26.9″ E), the village of Pusalotas, Pasvalys district, Panevezys county, Lithuania.

### 3.2. Collection of Samples

The bulk and rhizosphere soil samples were collected from the agricultural fields of the above-mentioned district. Fourteen days before the harvest of winter wheat, the bulk soil samples from the study-site were collected and characterized at the 0–20 cm depth layer for chemical and physical analyses. Each soil sample was sampled from seven different points in each agricultural field and mixed to obtain a composite sample. Three general samples (1 bulk soil sample × 3 fields) coded with the names JAK, BEI, and DEG and approximately 50 g fresh weight was poured into 150 mL sterile polypropylene containers and immediately transported to the laboratory for the bulk soil physicochemical analysis.

The *T. aestivum ARTIST C2* was used as a trap crop to isolate plant growth-promoting rhizobacteria from the rhizosphere soil. Seven crop plants were uprooted along with the soil from each field and immediately transported to the laboratory in sterile, zip-lock covers for further analysis. The large soil aggregates and non-rhizospheric soil were removed by strong shaking. For each of the seven plants, the soil that adhered to the roots was separately collected from the plant to get a rhizospheric soil sample. Three rhizosphere soil samples (1 rhizosphere soil sample × 3 field) were collected and coded with the same designations as bulk soil samples and were kept moist in the dark at 4 °C for the further isolation of microorganisms.

### 3.3. Physicochemical Analysis of Soil

Three soil samples were air-dried in the thermostatic incubator at around 20–25 °C temperature, sieved to pass a 2 mm sieve, and thoroughly homogenized. The information about the localization, geographic coordinates, altitude, and climate analyses are presented in Table 1. In a short description, soil pH was determined using a glass electrode pH meter at 1:2:5 soil–deionized water ratio [84]. The plant-available P and K were extracted using the Mehlich 3 (M3) extraction method and determined using the ammonium vanadate method and the amount determined using a spectrophotometer [85]. The organic carbon was determined by Walkley–Black sulfuric acid–dichromate by oxidation–reduction titration with ferrous ammonium sulfate [86]. The nitrogen (N) was determined using the Kjeldahl method by wet oxidation using concentrated sulfuric acid and digestion catalyst and the conversion of organic N to inorganic ammonium (NH_4_) [87]. The information about the chemical analyses is presented in Table 2.

### 3.4. Isolation of PGPR

One gram of soil from each rhizosphere sample was mixed with 9 mL of sterilized distilled water which gives a dilution of 1:10 (1 part soil in 10 parts solution) and then stirred at 130 RPM for 30 min. After the incubation period, 100 μL of the stock solution was transferred into a 1.5 mL Eppendorf tube containing 900 µL of sterile distilled water to obtain 10^−1^ dilution. The serial dilution method was repeated until 10^−5^ dilution. In the sequel, 100 μL of each suspension were spread on Luria Bertani (LB) Petri dishes which contained the following ingredients L^−1^: tryptone, 10 g; yeast extract, 5 g; NaCl, 10 g; and agar 15 g. The medium pH was adjusted to 7.0 ± 0.2 before autoclaving at 15 lbs pressure (121 °C) for 15 min and after the sterilization process poured into sterilized Petri dishes. The serial dilution suspensions were spread over the surface of an appropriate agar and incubated at 30 °C for 48–72 h. The morphologically different colonies which were considered to be plant growth-promoting rhizobacteria were repeatedly transferred and streaked several times on LB agar medium and incubated at 30 °C for 48–72 h to get single purified colonies. All the strains used in the experiments were cryo-preserved using 50% glycerol (*v*/*v*) at −80 °C for further use and deposited at the “Rhizospheric and Plant Growth Promoting Bacteria Culture Collection of *ARTIST C2*” in JSC Bioenergy LT, Panevezys, Lithuania.

### 3.5. Identification of PGPR

16S rRNA gene amplification was performed by using the bacterial-specific primers 27F (5′-AGAGTTTGATCCTGGCTCAG-3′) and 1492R (5′-GGTTACCTTGTTACGACTT-3′). The reaction consisted of the following: PCR master 12.5 μL, Forward primer 27F 1 μL, Reverse primer 1 μL, sterile distilled water 7.5 μL, and 3 μL DNA template. The amplification conditions used were as follows: initial denaturation for 5 min at 94 °C, 35 cycles of denaturation for 30 s at 94 °C, annealing for 30 s at 58 °C, extension for 30 s at 72 °C, and final extension for 7 min at 72 °C. The PCR products were visualized by the electrophoresis of 3 μL of the amplified DNA on 1% (*w*/*v*) horizontal agarose gel (SIGMA^®^) in TBE buffer (1.1 *w*/*v* Tris-HCl; 0.1% *w*/*v* Na_2_EDTA 2H_2_O; 0.55% *w*/*v* Boric acid), pre-stained with 3.5 μL of ethidium bromide. The gel was photographed under UV illumination with the Gel Doc (BIO-RAD) Software (Version 6.1, Hercules, CA, USA). The products with a single band were selected as suitable for purification. Phylogenetic trees were constructed based on 16S rRNA gene sequences by the Neighbor-Joining method using a distance algorithm with a bootstrap of 1000, with the Molecular Evolutionary Genetics Analysis (Version 11, Pennsylvania State University, University Park, PA, USA) software.

### 3.6. ZymoBIOMICS^®^ Targeted Sequencing

Bacterial 16S ribosomal RNA gene-targeted sequencing was performed using the *Quick*-16S^™^ NGS Library Prep Kit (Zymo Research, Irvine, CA, USA). The bacterial 16S primers amplified the V3-V4 region of the 16S rRNA gene. These primers have been custom-designed by Zymo Research to provide the best coverage of the 16S gene while maintaining high sensitivity. The sequencing library was prepared using an innovative library preparation process in which PCR reactions were performed in real-time PCR machines to control cycles and therefore limit PCR chimera formation. The final PCR products were quantified with qPCR fluorescence readings and pooled together based on equal molarity. The final pooled library was cleaned with the Select-a-Size DNA Clean & Concentrator™ (Zymo Research, Irvine, CA, USA), then quantified with TapeStation^®^ (Agilent Technologies, Santa Clara, CA, USA) and Qubit^®^ (Thermo Fisher Scientific, Waltham, WA, USA). The final library was sequenced on Illumina^®^ MiSeq™ with a v3 reagent kit (600 cycles). The sequencing was performed with a 10% PhiX spike-in. The taxonomy composition visualization assignment was performed using Uclust from the Qiime v.1.9.1 software with the Zymo Research Database.

### 3.7. Characterization of Selected PGPR

#### 3.7.1. NH_3_ Production

The ammonia production ability of the bacterial isolates in our study was detected in peptone water broth which contained the following ingredients L^−1^: peptone, 10 g and NaCl, 5 g; pH 7.2 ± 0.2. One milliliter of each bacterial suspension (1.0 × 10^8^ CFU mL^−1^) was incubated in 10 mL of peptone water broth and incubated at 130 RPM, 30 °C for 48 h. After the incubation period, 0.5 mL of Nessler’s reagent was added to each sample. A change in media color from yellow to brown was considered positive for NH_3_ production.

#### 3.7.2. HCN Production

The isolates were inoculated on the King’s B agar medium containing 4.4 g glycine L^−1^. Whatman filter paper No. 1 was saturated with 2% sodium carbonate in 0.5% picric acid solution and placed on the upper lids of the Petri plates and sealed with air-tight Parafilm. The plates were incubated at 30 °C for 2 days. A color change in the filter paper disc from deep yellow to reddish-brown was considered as an indication of HCN production.

#### 3.7.3. Siderofore Production

Siderophore production was conducted qualitatively on Chrome Azurole’s (CAS) agar medium. The PGPR isolates were inoculated as spots on the CAS agar medium surface and incubated at 30 °C for 7 days. After incubation, the development of a yellow–orange halo zone around the isolates indicated the production of siderophores.

#### 3.7.4. 1-Aminocyclopropane-1-Carboxylate Deaminase Production

The activity of the 1-aminocyclopropane-1-carboxylate (ACC) deaminase of the bacteria isolates was qualitatively determined by using the Dworkin Foster (DF) salt minimal medium with ammonium sulfate (NH_4_)_2_SO_4_ as a nitrogen source. The isolates were inoculated on the DF agar medium at 30 °C for 48 h, and then they were transferred to plates containing DF medium with 1-aminocyclopropane 1-carboxylate (ACC) as the only source of nitrogen instead of (NH_4_)_2_SO_4_. The plates were incubated at 30 °C for 7 days. The PGPR isolates that were able to grow in the medium of this composition were considered to have ACC deaminase activity.

#### 3.7.5. Catalase Activity

For the detection of catalase activity, a loop full of bacterial suspensions was placed on a separate, clean glass slide and a drop of 3% hydrogen peroxide (H_2_O_2_) was added to it. The effervescence of gas bubbles from the colony was considered to be a positive production of catalase enzymes.

#### 3.7.6. Nitrogen-Fixing Activity

The nitrogen-fixing activity was determined qualitatively on a glucose nitrogen-free mineral (GNFM) agar medium using bromothymol blue (BTB) as an indicator. The composition of the medium contained the following ingredients L^−1^: glucose, 10 g; K_2_HPO_4_, 1 g; MgSO_4_, 0.2 g; NaCl, 0.2 g; CaCO_3_, 1 g; NaMoO_4_, 0.005 g; FeSO_4_, 0.1 g; and agar, 20 g; the pH of the medium was adjusted to 7.0 ± 0.2 before autoclaving at 15 lbs pressure (121 °C) for 15 min. The bacterial suspension of each isolate was inoculated onto the GNFM agar surface and incubated at 28 °C for 5–7 days. The color changes indicated the strains with nitrogen-fixing activity.

#### 3.7.7. Potassium Solubilization Activity

The isolates were spot inoculated on Aleksandrow Agar medium consisting of the following ingredients L^−1^: dextrose, 5.0 g; CaCO_3_, 0.1 g; FeCl_3_, 0.005 g; MgSO_4_ × 7H_2_O, 0.5 g; Ca_3_PO_4_, 2.0 g; potassium alumina silicate, 2.0 g; agar, 20.0 g; and 500 µL of 0.5% bromothymol blue (BTB) as an indicator. The plates were incubated at 30 °C for 7 days. After spot inoculation, the ability of the bacterial isolates to dissolve potassium was analyzed based on the appearance of clear zones and a transformation in color from greenish blue to yellow.

#### 3.7.8. Phosphate Solubilization Activity

The phosphorus-solubilizing activity of isolates was determined qualitatively by using Pikovaskaya’s (PVK) agar medium that consisted of the following ingredients L^−1^: yeast extract, 0.50 g; dextrose, 10.00 g; Ca_3_(PO_4_)_2_, 5.00 g; (NH_4_)_2_SO_4_, 0.500 g; KCl, 0.20 g; MgSO_4_, 0.10 g; MnSO_4_, 0.0001 g; FeSO_4_, 0.0001 g; and agar, 15.00 g; the pH of the medium was adjusted to 7.0 ± 0.2 before autoclaving at 15 lbs pressure (121 °C) for 15 min. Each isolate was placed on the PVK agar plates with the help of a loop, and then the plates were incubated at 30 °C for 7 days. The transparent halo zone formation around the isolated colonies after the incubation period indicated a positive result for phosphate solubilizing activity.

### 3.8. Quantitative Estimation of Phosphate Solubilization Activity

The quantitative estimation of inorganic phosphate solubilization was performed in the modified NBRIP broth medium. The tricalcium phosphate in the nutrient medium was replaced by natural rock phosphate (RP) from Morocco as the only source of insoluble phosphorus. Pre-inoculum cultures were prepared earlier by inoculating phosphate-solubilizing isolates in LB broth and incubated at 130 RPM, 30 °C for 16–18 h. The cell density of each inoculum was adjusted from 10^7^ to 10^8^ CFUmL^−1^. One mL of the prepared isolate suspension (or a mix of two or three isolates in equal parts) was inoculated into the 100 mL liquid-modified NBRIP medium with RP and incubated at 130 RPM, 30 °C for 30 days. The modified NBRIP broth medium without any inoculant was prepared as the control sample. After the incubation period, all the suspensions were centrifuged at 10,000 RPM for 30 min to remove insoluble sediment and cell biomass. The dissolved phosphorus content in filtrates was determined using the molybdenum blue method described by Salem F.B. [88] and conducted by VR-2000 spectrophotometer by determining the optical density at 850 nm (OD_850_). All the values of the dissolved phosphate concentrations of the samples were measured in triplicates.

### 3.9. Analysis of Organic Acids by HPLC Dionex Ultimate 3000-4

Organic acids identification and quantification were performed using an HPLC Dionex Ultimate 3000-4 (International Equipment Trading Ltd., Mundelein, IL, USA) equipped with a column oven and an integrated Aminex HPX-87H column (300 × 7.8 mm) (Bio-Rad, Hercules, CA, USA). The samples were centrifuged at 4200 RPM for 10 min. The resulting supernatant was diluted with acetonitrile (ratio 1:1). The lower fraction was collected and diluted at a ratio of 1:3 with water. The solution was filtered via the C18 SPE column (Bond Elut-C18, Agilent) and through 0.45 μm filters (GHP Acrodisc Pall). The sample analysis was performed on a Dionex Ultimate 3000-4 chromatograph equipped with an Aminex HPX-87H column (300 × 7.8 mm; Bio-Rad) maintained at 60 °C using 5 mM H_2_SO_4_ eluent (prepared from 0.1 M H_2_SO_4_; Supelco). Mobile phase flow rate 0.4 mL min, injection volume 20 μL. An RI (refractive index) detector was used for detection. The measurements were carried out in triplicate. All the data were processed using the MassHunter 7.00 software (Agilent Technologies, Waldbronn, Germany). The concentration of organic acids was calculated from standard curves.

### 3.10. Analysis of Phytohormones by Liquid Chromatography Time-of-Flight Mass Spectrometry

The method of sample preparation was adapted from Castillo et al. [89]. The samples were centrifuged at 13,000 RPM for 10 min, and then the supernatant aliquot (3 mL) was adjusted to pH 2–3 with 3 M HCl. The resulting mixture was extracted with 3 mL ethyl acetate by shaking for 5 min and centrifuged at 4200 RPM for 5 min. The extraction was repeated three times. The combined ethyl acetate fraction was evaporated to dryness under a nitrogen stream at 40 °C and dissolved in 1.0 mL of 20% methanol in water containing 0.1% formic acid. The resulting solution was filtered through a 0.45 µm filter and analyzed by LC-TOF/MS. The identification and quantification of indole-3-acetic acid, zeatin, gibberellic acid, and abscisic acid were performed using an Agilent 6230 TOF LC/MS system (Agilent Technologies, Waldbronn, Germany) equipped with electrospray ionization (ESI). Kinetex C18 column (3.0 × 100 mm, 2.6 µm) was used for the chromatographic separation at 40 °C. The mobile phase composed of 0.1% aqueous formic acid (A) and 0.1% formic acid in methanol (B). Gradient elution was conducted according to the following conditions: 0 min, 10% B; 2 min, 10% B; 3 min, 30% B; 4.5 min, 30% B; 5.5 min, 50% B; 8.5 min, 50% B; 14.5 min, 70% B; 16 min, 98% B; 18 min, 98% B; 19 min, 10% B; and 23 min, 10% B. The flow rate was 0.2 mL min^−1^ and the volume of injection was 20 µL. The mass spectrometer settings were as follows: drying gas flow, 12 L min^−1^; drying gas temperature, 320 °C; nebulizer pressure, 40 psi; capillary voltage, 3500 V; and fragmentor voltage, 130 V. ESI-MS was used in positive ionization mode and the full scan mass range was 50–1000 m/z. The calibration was carried out using internal reference masses 121.0509 m/z and 922.0098 m/z. Each sample analysis was performed in triplicate. The MassHunter 7.00 software (Agilent Technologies, Waldbronn, Germany) was used to analyze the data. The content of abscisic acid, zeatin, gibberellic acid, and indole-3-acetic acid was determined from standard curves.

### 3.11. Co-Inoculation Test in Non-Sterile Soils through Pot Experiment

The *T. aestivum* (*ARTIST C2* variety) seeds were obtained from the Krekenava subdivision, the village of Pusalotas, Pasvalys district, Panevezys county, Lithuania. The seeds were surface sterilized with 2% of sodium hypochlorite for 5 min followed by 70% of ethanol for 5 min. Another rinse with sterile water was repeated five times and the final rinse water was used to assess the sterilization process. The pre-selected healthy seeds of uniform size were then planted in plastic pots (volume = 2.5 L) that contained two kilograms of the non-sterilized mixture of soil, vermiculite, peat moss, and perlite (ratio: 5,1,1,1 *v*/*v*). One milliliter of two-days-old isolate suspension was inoculated in the treatment pot 7 days after seeds planting. For the co-inoculation treatments, a cocktail consisting of two or three isolates was prepared in the ratio of 1:1 or 1:1:1, and 1 mL of the mixture was inoculated per pot 7 days after seed planting. As for the uninoculated treatment, which represented the control, only 1 mL of LB was supplemented. After 14 days of cultivation, 1 mL of the bacterial suspension was re-inoculated in bacterial inoculation treatments. The pots were watered regularly to maintain the soil at field capacity. Each treatment consisted of five replicates. The seedlings were maintained at 28 °C and under a 16:8 day–night photoperiod in a growth chamber and the crops were grown under greenhouse conditions for about 55 days. After 55 days of cultivation, the growth of wheat including root length, shoot length, and the number of leaves; the shoot and root fresh weights; and shoot and root dry weights (drying at 70 °C) were measured. The K, Ca, Mg, Na, NH_4_, NO_3_, N in Nitrate, N—Total Nitrogen, Cl, S, P, Si, Fe, Mn, Zn, B, Cu, Mo, and Al contents of the plant materials were sent to the research and test center specializing in plant sap analysis (NovaCropControl, Nijverheidsweg 30, 5061 KL Oisterwijk, the Netherlands).

### 3.12. Statistical Analysis

All of the measurements were made in triplicate. The results were subjected to an analysis of variance (ANOVA), and the means were separated by the Tukey test (*p* ≤ 0.05) by using the GraphPad Prism 10 software (GraphPad Software, San Diago, CA, USA).

## 4. Conclusions

The research objectives were to select three different agricultural areas with the same crop rotation technology but with different soil pH values: neutral (pH 7.08), slightly acidic (pH 6.11), and slightly alkaline (pH 8.00). *T. aestivum* (*ARTIST C2* variety) was cultivated in these fields for about twelve months (from 08/2021 to 08/2022). In this study, we isolated the rhizosphere microorganisms (three from each agricultural area) of which one each belonged to the *Bacillus*, *Pseudomonas,* and *Streptomyces* genera. Isolated microorganisms were used as single inoculation or mixed with each other to obtain the best compatible SynComs from three various genera. The effective P-solubilizing SynComs were detected in IJAK-27+44+91, IIBEI-32+40, III-DEG-45+41, and IIIDEG-45+41+72 and demonstrated its potential as biofertilizers, which were capable of producing organic lactic, acetic, gluconic, malic, oxalic and citric acids and as well as a range of phytohormones such as indole-3-acetic acid, zeatin, gibberellic acid, and abscisic acid. We also demonstrated that the designed SynComs were able to effectively colonize the rhizosphere winter wheat plants and were a good root colonizer. Also, we found that SynCom consisting of three different genera was not the best option in all cases. In the first treatment, the best results were obtained with the SynCom composed of *Bacillus*, *Pseudomonas,* and *Streptomyces* genus. In contrast, the second analysis showed that the best SynCom was obtained only by *Bacillus* and *Pseudomonas* genera, and in a third trial, no significant differences were observed between the SynCom of *Bacillus* and *Pseudomonas* or the SynCom of *Bacillus*, *Pseudomonas*, and *Streptomyces* genera. However, in all the cases, SynComs showed a superior result than the single microorganism as biofertilizers. This leads to the conclusion that it is very important to select the appropriate species that will demonstrate the most optimal results. Furthermore, efforts should be made to elucidate the relationships and mechanisms involved in the association between each microorganism. Future experiments need to be conducted to validate the effective capability of those PGPRs in real field conditions. The optimization of the processes of microorganism selection and their interactions in the rhizosphere are stages to improve the development of efficient SynCom inoculants for the agrobiotechnology sector.

## Figures and Tables

**Table 1:** Field localization, geographic coordinates, altitudes, and climatic characteristics of the sites of soil source.

Field	Coordinates ^1^	Altitude (m) ^2^	Climate Type ^3^	Average Annual Lower Temperature (°C)	Average Annual Higher Temperature (°C)	Average Annual Rainfall (mm)
Jakuboniai/JAK	55°54′00.3″ N 24°06′44.3″ E	54	*Dfb*	−6.0	23.0	65.1
Beicarke/BEI	55°55′53.6″ N 24°16′46.2″ E	41				
Deglenai/DEG	55°56′17.8″ N 24°08′26.9″ E	48				

^1^ Latitude, longitude; ^2^ Above sea level; ^3^ According to Köppen–Geiger classification.

**Table 2:** Chemical properties of soil samples (0–0.20 m) from experimental sites.

Field	Depth (cm)	pH Value (H_2_O)	Available P (mg Kg^−1^)	Available K (mg Kg^−1^)	Organic Carbon (%)	N-NO_3_ (mg Kg^−1^)	N-NH_4_ (mg Kg^−1^)	N_min_ (mg Kg^−1^)
JAK	0–20	7.08	115	129	1.19	1.43	2.35	3.77
BEI		6.11	199	141	0.95	8.32	0.74	9.06
DEG		8.00	159	265	2.12	15.20	0.50	15.70

**Table 3:** Molecular identification of plant growth promoting rhizobacteria by 16S rDNA sequencing (see Supplementary Figures S1 and S2).

Sequence ID	Blast-Related Sequence	Strain	The Number Assigned to the Sequence	Sequence Similarity (%)	Mismatched Nucleotides/Total Nucleotides	Sequence Coverage (%)
IJAK-27	*Bacillus toyonensis*	BCT-7112	CP006863	99.71223022	4/1390	100
IJAK-44	*Pseudomonas bijieensis*	L22-9	MT835388	99.78355	3/1386	100
IJAK-91	*Streptomyces gardneri*	NBRC 12865	AB249908	100	0/1360	100
IIBEI-32	*Bacillus aryabhattai*	B8W22	EF114313	100	0/1400	100
IIBEI-40	*Pseudomonas helmanticensis*	OHA11	HG940537	99.12	12/1376	100
IIBEI-22	*Streptomyces anulatus*	NRRL B-2000	DQ026637	99.57	6/1421	100
IIIDEG-45	*Bacillus tequilensis*	KCTC 13622	AYTO01000043	99.85	2/1410	100
IIIDEG-41	*Pseudomonas granadensis*	LMG 27940	LT629778	99.34783	9/1380	100
IIIDEG-72	*Streptomyces badius*	NRRL B-2567	AY999783	99.50	7/1421	100

**Table 4:** Plant growth promoting of the bacteria isolated from root rhizosphere of *T. aestivum*.

Strain	Ammonia (NH_3_) Production	HCN Production	Siderophore Production	ACC Deaminase Production	Catalase Activity	Nitrogen-Fixing Activity	Potassium Solubilisation	Phosphate Solubilisation
IJAK-27	+++	+++	++	+++	+++	++	+	++
IJAK-44	+	++	+++	+++	++	++	++	+++
IJAK-91	+	+	++	++	+	++	+	+
IIBEI-32	+++	++	++	+++	++	+	+	++
IIBEI-40	+	+	++	++	+	+	+	+++
IIBEI-22	−	−	++	+++	+	+	+	+
IIIDEG-45	+++	+++	++	++	+++	+++	+	++
IIIDEG-41	+	+	+++	++	++	+	++	+++
IIIDEG-72	−	−	+	++	+	+	+	+

(+++) highly positive result, (++) moderately result, (+) weakly positive result, and (−) negative result.

**Table 5:** Organic acid detection in single or co-inoculation supernatants in NBRIP medium using HPLC Dionex Ultimate 3000-4.

Treatment	Concentration, µg mL^−1^ (Average from Triplicates ± SD)					
	Lactic Acid	Acetic Acid	Gluconic Acid	Malic Acid	Oxalic Acid	Citric Acid
Control	8.0 ± 1.0	5.0 ± 0.2	ND †	ND	ND	ND
IJAK-27	62.0 ± 5.0	6.5 ± 0.2	19.0 ± 2.0	25.0 ± 2.0	0.6 ± 0.1	20.0 ± 2.0
IJAK-44	240.0 ± 6.0	16.0 ± 2.0	59.0 ± 4.0	62.0 ± 1.0	1.0 ± 0.3	10.0 ± 1.0
IJAK-91	10.5 ± 1.0	13.0 ± 0.7	25.0 ± 2.0	6.0 ± 0.3	ND	ND
IJAK-27+44	333.5 ± 8.0	33.0 ± 2.0	83.0 ± 2.0	77.5 ± 4.0	1.8 ± 0.2	70.0 ± 4.0
IJAK-27+91	101.0 ± 16.0	15.0 ± 3.0	34.0 ± 3.0	21.5 ± 1.0	0.8 ± 0.2	27.0 ± 3.0
IJAK-44+91	222.0 ± 4.0	28.0 ± 1.0	42.0 ± 3.0	64.0 ± 1.0	1.1 ± 0.2	15.0 ± 3.0
IJAK-27+44+91	567.0 ± 13.0	35.0 ± 2.0	108.0 ± 5.0	140.0 ± 7.0	1.2 ± 0.2	33.5 ± 6.0
IIBEI-32	105.0 ± 1.0	13.0 ± 0.7	87.0 ± 13.0	6.0 ± 0.3	1.1 ± 0.3	13.5 ± 0.6
IIBEI-40	267.0 ± 6.0	11.5 ± 2.0	88.5 ± 5.0	21.0 ± 3.0	1.0 ± 0.3	7.0 ± 0.2
IIBEI-22	26.0 ± 4.0	ND	18.5 ± 2.0	14.0 ± 1.0	ND	ND
IIBEI-32+40	399.0 ± 7.0	25.5 ± 4.0	180.0 ± 7.0	105.0 ± 5.0	1.0 ± 0.5	27.0 ± 0.2
IIBEI-32+22	107.0 ± 2.0	10.0 ± 1.0	78.0 ± 8.0	2.0 ± 0.2	0.5 ± 0.1	4.5 ± 0.5
IIBEI-40+22	200.0 ± 4.0	17.0 ± 2.0	140.0 ± 4.0	92.0 ± 2.0	1.5 ± 0.2	10.0 ± 0.5
IIBEI-32+40+22	186.0 ± 6.0	12.5 ± 2.0	139.0 ± 7.0	66.0 ± 4.0	0.9 ± 0.3	5.0 ± 1.0
IIIDEG-45	201.0 ± 2.0	15.0 ± 2.0	13.0 ± 1.0	4.0 ± 1.0	0.2 ± 0.1	1.0 ± 0.1
IIIDEG-41	213.0 ± 5.0	17.0 ± 2.0	37.0 ± 4.0	4.0 ± 0.5	0.3 ± 0.1	1.0 ± 0.3
IIIDEG-72	26.0 ± 2.0	3.0 ± 0.5	42.5 ± 3.0	ND	ND	ND
IIIDEG-45+41	455.0 ± 15.0	35.0 ± 2.0	88.0 ± 3.0	11.0 ± 3.0	0.7 ± 0.2	5.0 ± 1.0
IIIDEG-45+72	222.0 ± 6.0	15.0 ± 2.0	55.0 ± 4.0	3.0 ± 0.5	ND	ND
IIIDEG-41+72	245. ± 7.0	34.0 ± 2.0	7.0 ± 1.0	2.0 ± 0.5	0.2 ± 0.1	0.2 ± 0.1
IIIDEG-45+41+72	405.0 ± 7.0	33.5 ± 3.0	101.5 ± 2.0	6.0 ± 2.0	1.3± 0.1	9.0 ± 0.8

† ND: not detectable.

**Table 6:** Phytohormones detection in four different SynCom supernatants using LC-TOF/MS.

Treatment	Concentration, µg mL^−1^ (Average from Triplicates ± SD)			
	Indole-3-Acetic Acid	Zeatin	Gibberellic Acid	Abscisic Acid
IJAK-27+44+91	6.600 ± 0.500 a	0.150 ± 0.020 b	3.400 ± 0.400 a	0.250 ± 0.020 a
IIBEI-32+40	3.500 ± 0.300 b	0.160 ± 0.010 b	1.240 ± 0.100 d	0.136 ± 0.003 b
IIIDEG-45+41	0.382 ± 0.005 c	1.100 ± 0.200 a	1.700 ± 0.300 c	0.271 ± 0.006 a
IIIDEG-45+41+72	0.348 ± 0.020 d	0.159 ± 0.011 b	2.250 ± 0.040 b	0.271 ± 0.012 a

Data represented as mean (n = 6) ± standard deviation. Different letters indicate significant differences (*p* < 0.05) in one-way ANOVA.

**Table 7:** Effect of co-inoculation with SynComs on the *T. aestivum* pot experiments.

Growth Indexes	CON1 †	CON2 ‡	IJAK-27+44+91	IIBEI-32+40	IIIDEG-45+41	IIIDEG-45+41+72
Root length (cm)	25.83 c	27.33 bc	36.00 a	35.17 a	34.33 a	33.83 ab
Shoot length (cm)	51.67 b	55.83 ab	63.50 a	63.17 a	62.00 ab	61.50 ab
Number of leaves	6.28 b	7.90 ab	8.30 a	7.96 a	7.86 ab	7.90 ab
Shoot fresh weight (g)	6.15 b	6.92 ab	9.60 a	9.62 a	8.23 ab	7.85 ab
Root fresh weight (g)	7.05 b	8.68 ab	10.93 a	10.73 a	8.85 ab	8.80 ab
Shoot dry weight (g)	0.53 c	0.57 bc	0.87 ab	0.90 a	0.71 ac	0.69 ac
Root dry weight (g)	0.54 d	0.66 cd	0.97 a	0.89 ab	0.83 ac	0.77 bc

† CON1: uninoculated treatment. ‡ CON2: uninoculated treatment with superphosphate (NPK 0-19-0). Data represented as mean (n = 6) ± standard deviation. Different letters indicate significant differences (*p* < 0.05) in one-way ANOVA.

**Table 8:** Effects of bacterial co-inoculation on the nutrient contents of *T. aestivum* plants grown in pot experiments.

Growth Indexes	CON2	CON1	IJAK-27+44+91	IIBEI-32+40	IIIDEG-45+41	IIIDEG-45+41+72
**Sugars, %**	1.7	1.3	2.0	2.2	2.2	1.9
**pH**	5.8	5.8	5.8	5.8	5.8	5.9
**EC, mS/cm**	10.1	9.2	10.1	9.9	9.9	9.6
**K, ppm**	4451	3989	4470	4278	4879	4367
**Ca, ppm**	1073	876	1390	1634	1112	1328
**Mg, ppm**	375	334	427	410	371	365
**Na, ppm**	38	19	21	35	33	33
**NH_4_, ppm**	49	41	52	45	57	51
**NO_3,_ ppm**	123	25	26	42	74	166
**N in Nitrate, ppm**	28	6	9	6	17	38
**N—Total Nitrogen, ppm**	1713	1324	1522	1684	1975	1690
**Cl, ppm**	802	570	665	576	672	644
**S, ppm**	165	151	208	191	182	164
**P, ppm**	633	429	638	635	604	573
**Si, ppm**	13.0	13.3	23.8	26.3	19.9	19.4
**Fe, ppm**	1.99	1.90	2.21	2.7	2.75	2.22
**Mn, ppm**	7.12	6.27	7.64	6.85	5.53	7.54
**Zn, ppm**	2.03	1.31	2.00	2.3	3.6	2.04
**B, ppm**	2.27	1.93	2.60	2.4	2.47	2.36
**Cu, ppm**	0.29	0.19	0.27	0.29	0.31	0.37
**Mo, ppm**	0.10	<0.05	0.08	0.10	0.10	0.09
**Al, ppm**	0.88	<0.05	0.82	1.01	1.05	0.88

## Data Availability

The raw data supporting the conclusions of this article will be made available upon request to the corresponding authors.

## Supplementary Materials

The following supporting information can be downloaded at: https://www.mdpi.com/article/10.3390/plants13121716/s1, Figure S1: Evolutionary relationships of three different taxa (*Bacillus* spp. (**A**); *Pseudomonas* spp. (**B**), and *Streptomyces* spp. (**C**)). The evolutionary history was inferred using the Neighbor-Joining method. The bootstrap consensus tree inferred from 1000 replicates represents the evolutionary history of the taxa analyzed. Branches corresponding to partitions reproduced in less than 50% of bootstrap replicates are collapsed. The percentage of replicate trees in which the associated taxa clustered together in the bootstrap test (1000 replicates) are shown next to the branches. The evolutionary distances were computed using the Maximum Composite Likelihood method and are in the units of the number of base substitutions per site. This analysis involved 15 nucleotide sequences. All ambiguous positions were removed for each sequence pair (pairwise deletion option). There were a total of 1589 positions in the final dataset. Evolutionary analyses were conducted in MEGA11 software; Figure S2: Cells morphology of the four SynComs: IJAK-27+44+91 (**A**), IIBEI-32+40 (**B**), IIIDEG-45+41 (**C**), and IIIDEG-45+41+72 (**D**).
