# Supplementary material for: The Co-Inoculation Effect on Triticum aestivum Growth with Synthetic Microbial Communities (SynComs) and Their Potential in Agrobiotechnology
Source: Plants (Basel). 2024 Jun 20;13(12):1716. doi: 10.3390/plants13121716 (PMC11207813; doi:10.3390/plants13121716)
Supplement: Supplementary file 1 [file plants-13-01716-s001.zip › plants-3022531-supplementary.pdf]

Supplementary materials

The Co-inoculation Effect on Triticum aestivum Growth with Synthetic Microbial Communities (SynCom's) and their Potential in Agrobiotechnology

Raimonda Mažylytė[1*], Jurgita Kailiuvienė[2], Edita Mažonienė[2], Liana Orola[3], Justina Kaziūnienė[4], Kamilė Mažylytė[1], Eglė Lastauskienė[1] and Audrius Gegeckas[1]

[1] Life Sciences Center, Institute of Biosciences, Vilnius University, LT-10257 Vilnius, Lithuania; raimonda.mazylyte@gmc.stud.vu.lt, kamile.mazylyte@gmc.stud.vu.lt, egle.lastauskiene@gf.vu.lt, audrius.gegeckas@gf.vu.lt
[2] Roquette Amilina, LT-35101 Panevezys, Lithuania; jurgita.kailiuviene@roquette.com, edita.mazoniene@roquette.com
[3] Faculty of Chemistry, University of Latvia, LV-1004 Riga, Latvia; liana.orola@lu.lv
[4] Institute of Agriculture, Lithuanian Research Centre for Agriculture and Forestry, LT-58344 Akademija, Lithuania; justina.kaziuniene@lammc.lt
* Correspondence: raimonda.mazylyte@gmc.stud.vu.lt

A

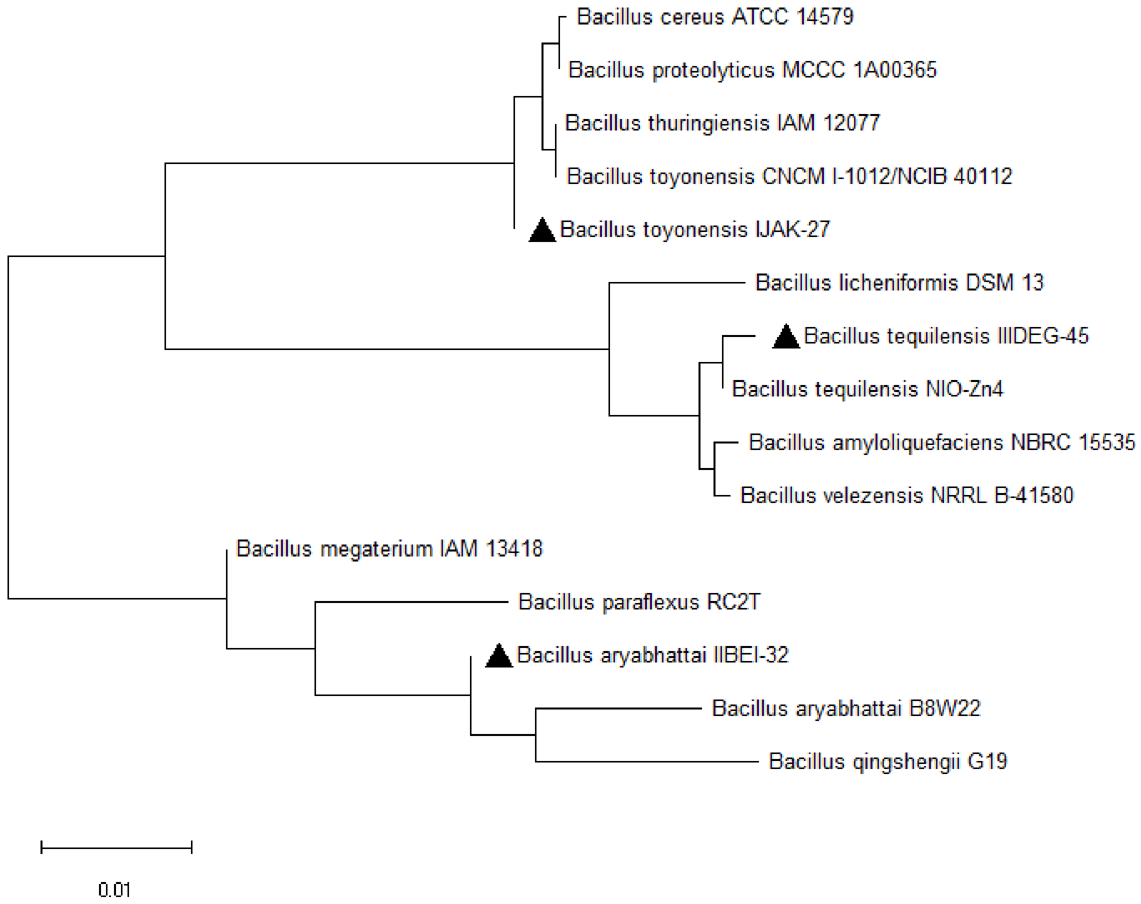

B

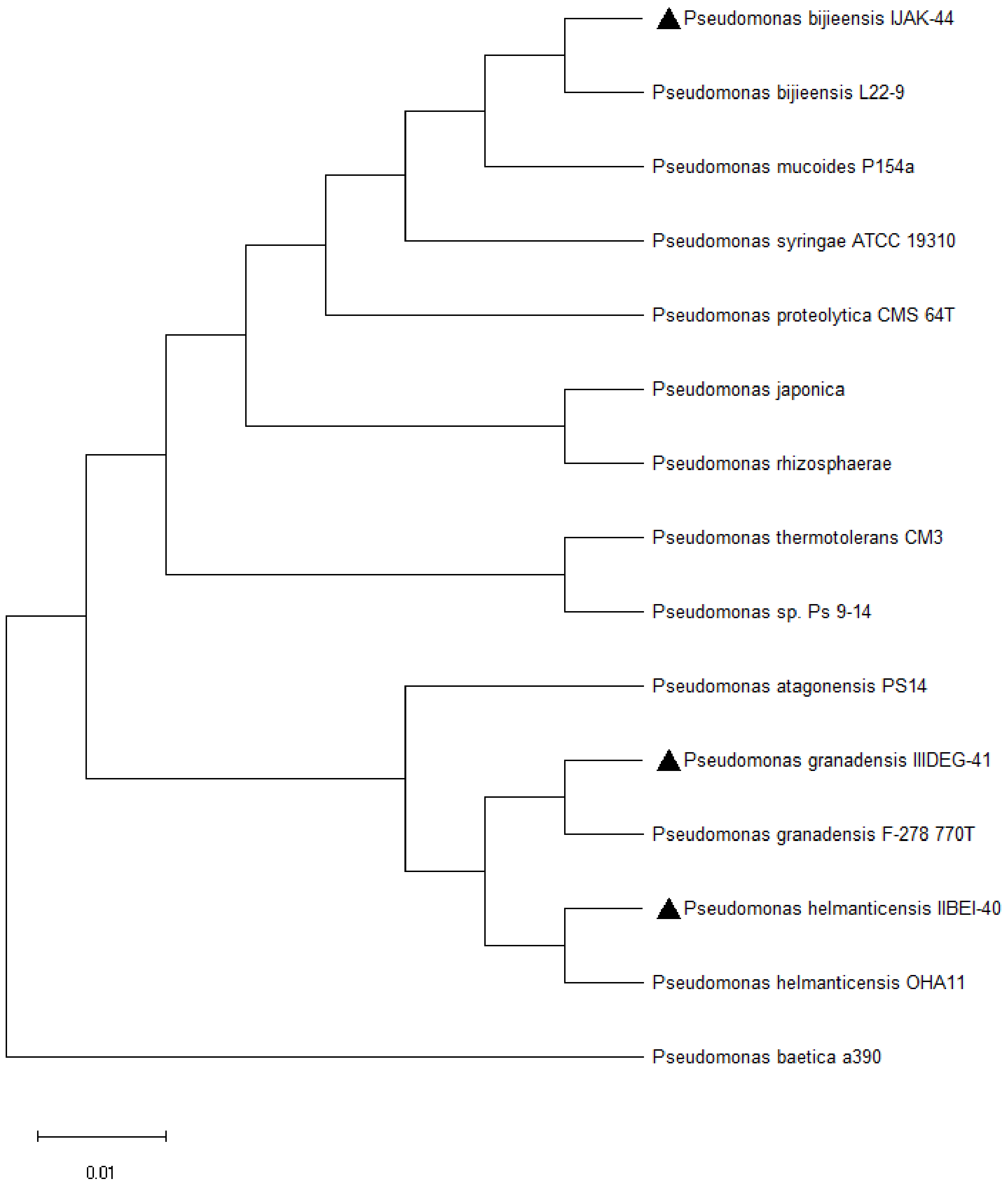

Pseudomonas bijieensis IJAK-44
Pseudomonas bijieensis L22-9
Pseudomonas mucoides P154a
Pseudomonas syringae ATCC 19310
Pseudomonas proteolytica CMS 64T
Pseudomonas japonica
Pseudomonas rhizosphaerae
Pseudomonas thermotolerans CM3
Pseudomonas sp. Ps 9-14
Pseudomonas atagonensis PS14
Pseudomonas granadensis IIIDEG-41
Pseudomonas granadensis F-278 770T
Pseudomonas helmanticensis IIBEI-40
Pseudomonas helmanticensis OHA11
Pseudomonas baetica a390
0.01

C

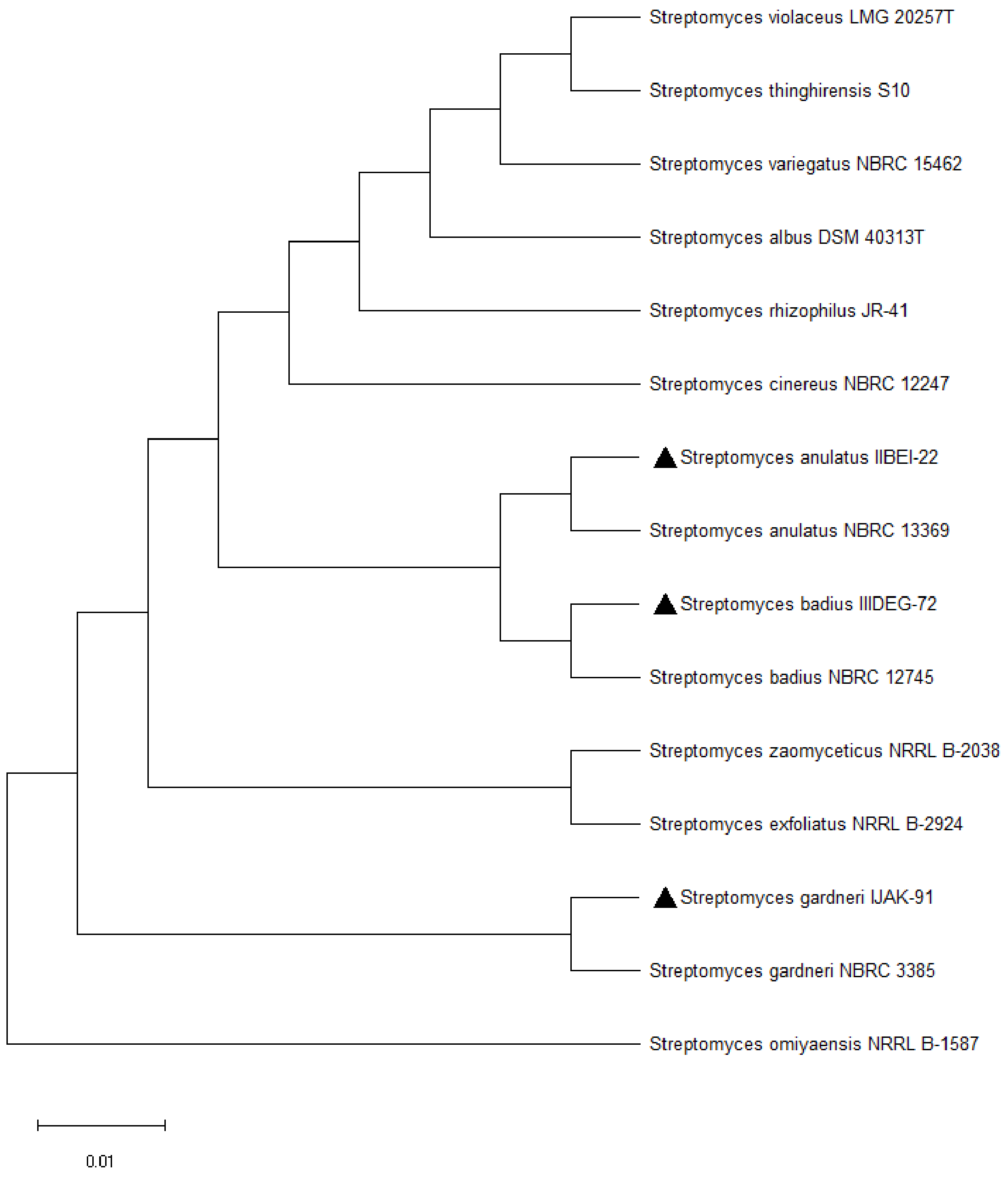

Figure S1. Evolutionary relationships of three different taxa (*Bacillus* spp. (**A**); *Pseudomonas* spp. (**B**), and *Streptomyces* spp. (**C**)). The evolutionary history was inferred using the Neighbor-Joining method. The bootstrap consensus tree inferred from 1000 replicates represents the evolutionary history of the taxa analyzed. Branches corresponding to partitions reproduced in less than 50% of bootstrap replicates are collapsed. The percentage of replicate trees in which the associated taxa clustered together in the bootstrap test (1000 replicates) are shown next to the branches. The evolutionary distances were computed using the Maximum Composite Likelihood method and are in the units of the number of base substitutions per site. This analysis involved 15 nucleotide sequences. All ambiguous positions were removed for each sequence pair (pairwise deletion option). There were a total of 1589 positions in the final dataset. Evolutionary analyses were conducted in MEGA11 software.

A

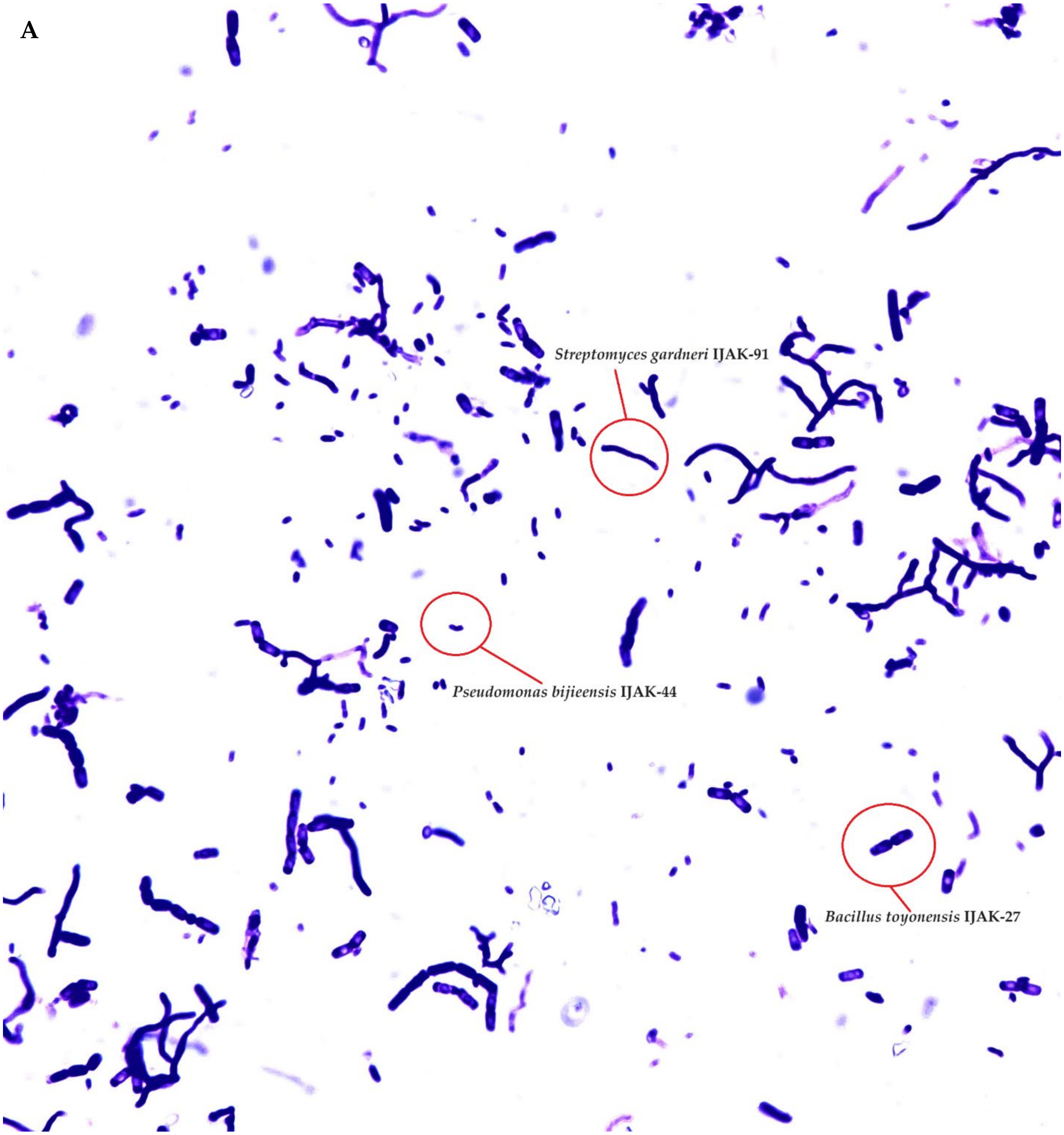

Streptomyces gardneri IJAK-91
Pseudomonas bijieensis IJAK-44
Bacillus toyonensis IJAK-27

B

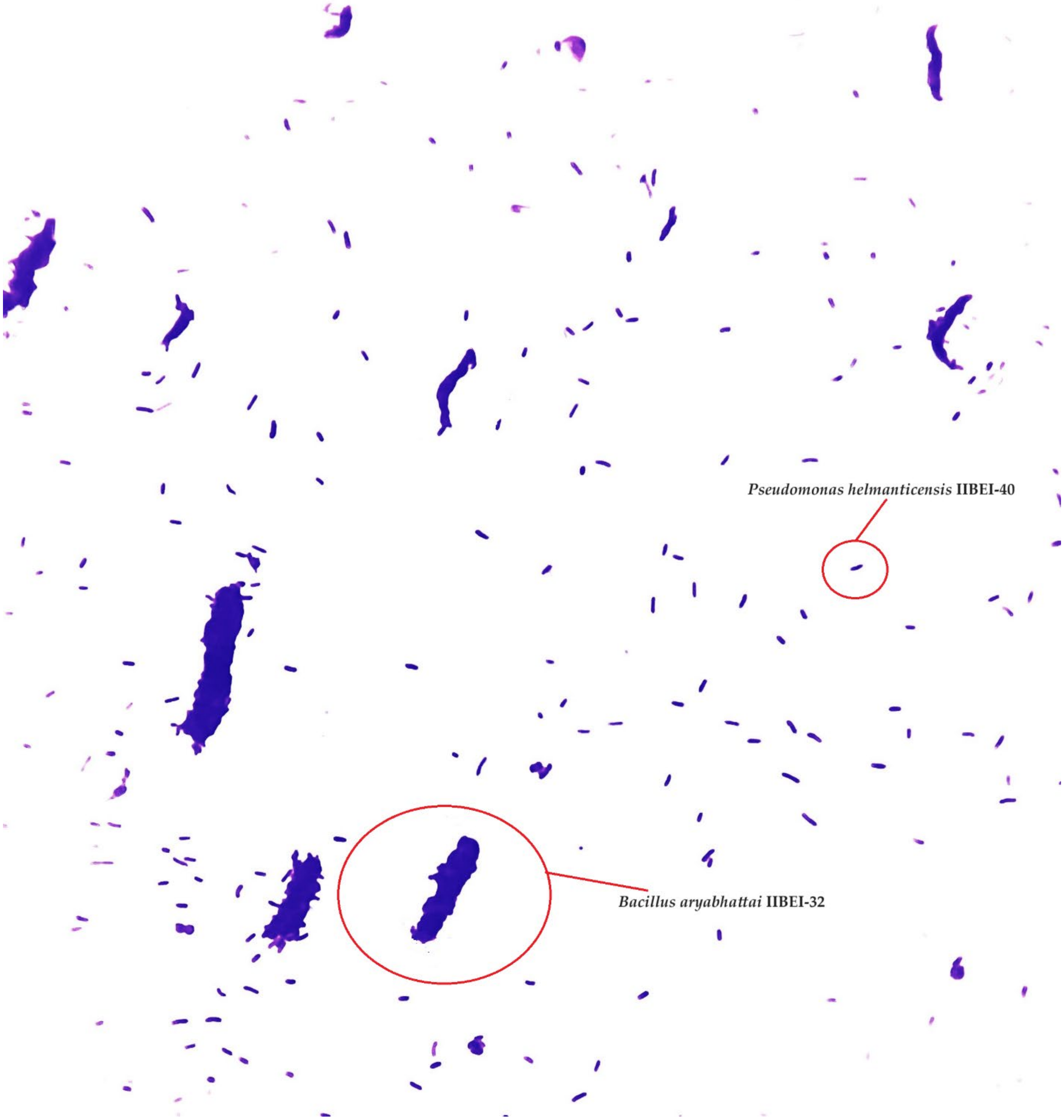
Pseudomonas helmanticensis IIBEI-40
Bacillus aryabhattai IIBEI-32

C

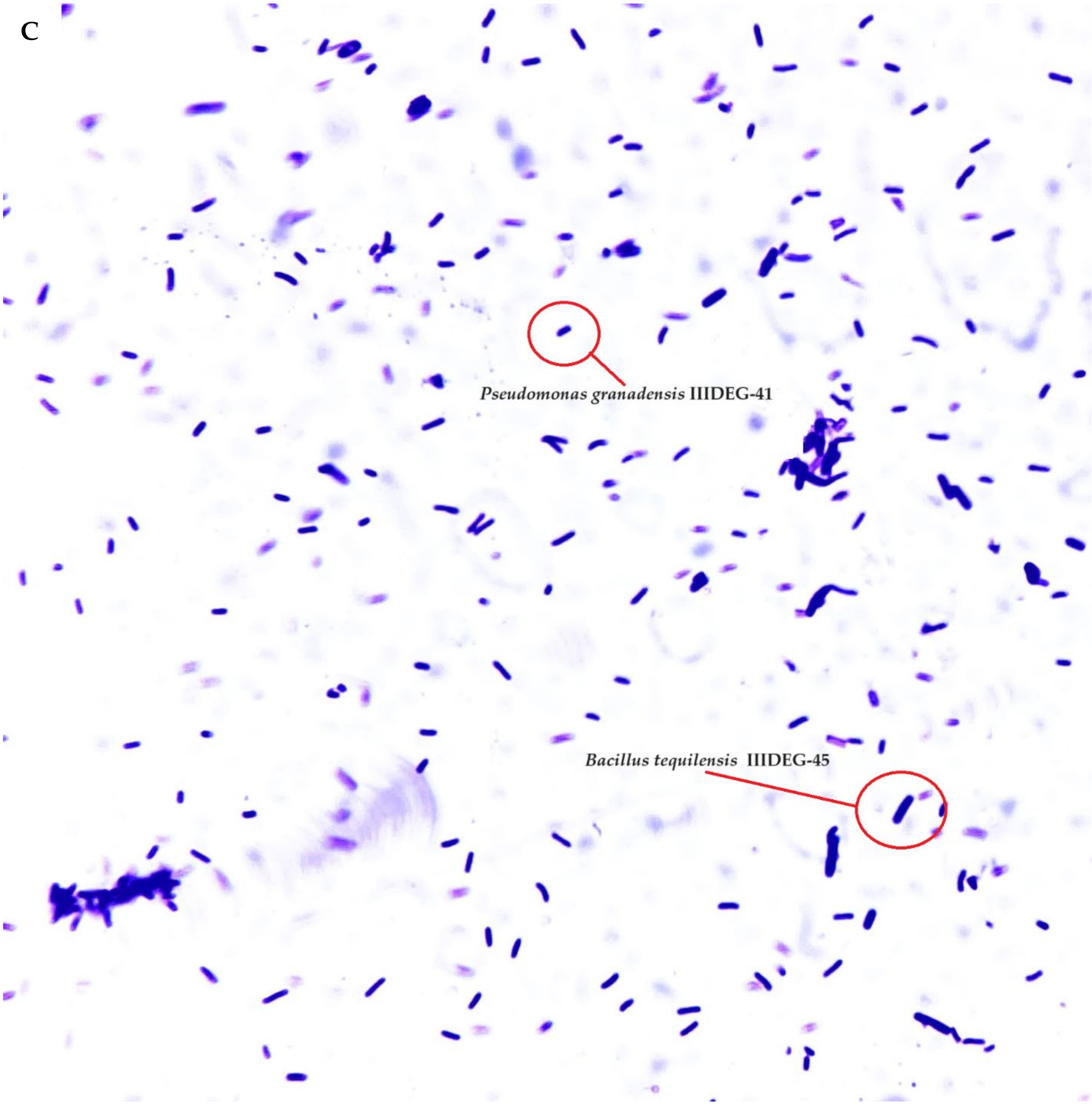

Pseudomonas granadensis IIIDEG-41
Bacillus tequilensis IIIDEG-45

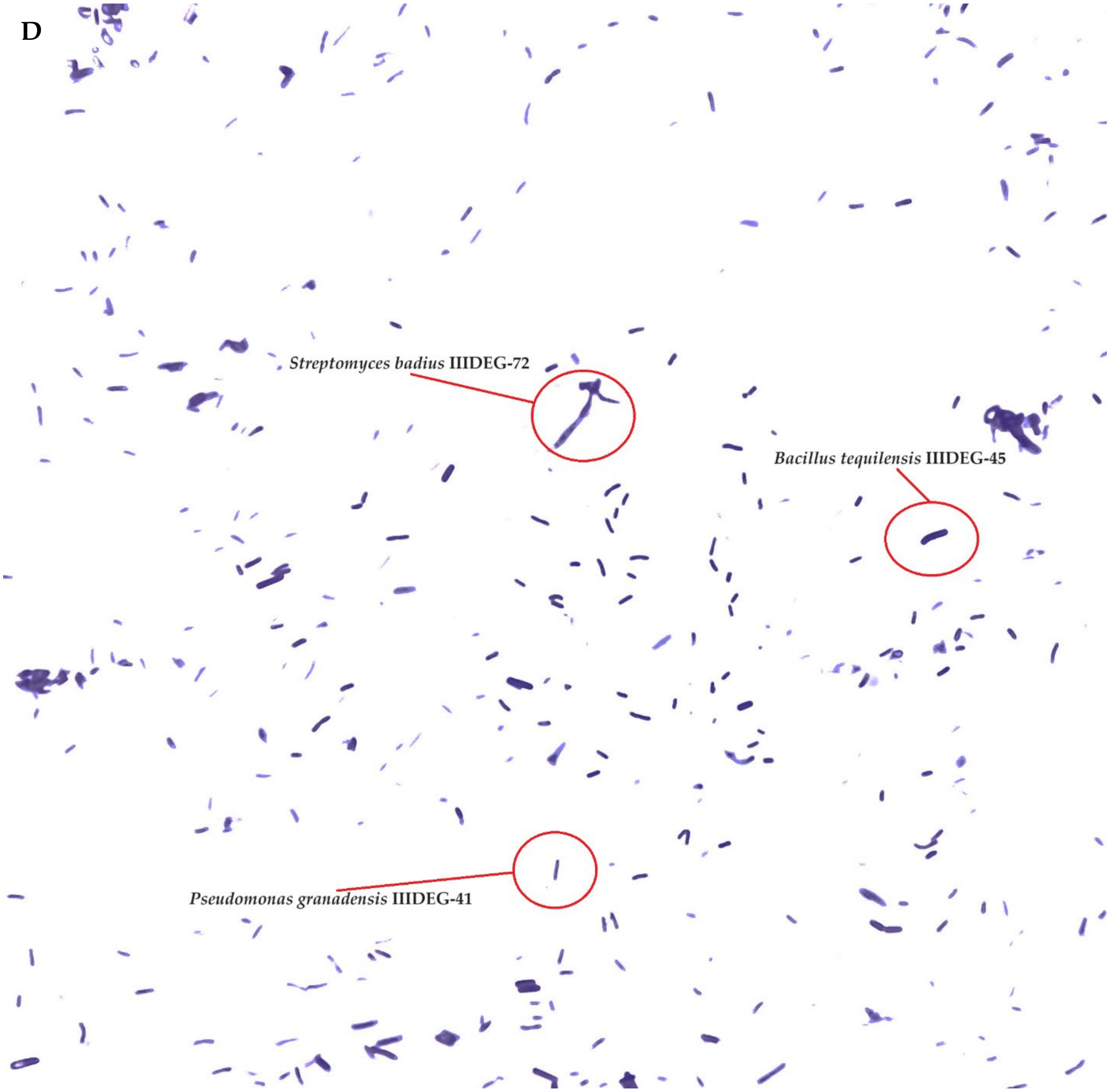

Figure S2. Cells morphology of the four SynComs: IJAK-27+44+91 (**A**), IIBEI-32+40 (**B**), IIIDEG-45+41 (**C**), and IIIDEG-45+41+72 (**D**).
